# A quorum sensing-independent path to stumpy development in *Trypanosoma brucei*

**DOI:** 10.1371/journal.ppat.1006324

**Published:** 2017-04-10

**Authors:** Henriette Zimmermann, Ines Subota, Christopher Batram, Susanne Kramer, Christian J. Janzen, Nicola G. Jones, Markus Engstler

**Affiliations:** Department of Cell and Developmental Biology, Biocenter, University of Würzburg, Würzburg, Germany; University of Massachusetts Amherst, UNITED STATES

## Abstract

For persistent infections of the mammalian host, African trypanosomes limit their population size by quorum sensing of the parasite-excreted stumpy induction factor (SIF), which induces development to the tsetse-infective stumpy stage. We found that besides this cell density-dependent mechanism, there exists a second path to the stumpy stage that is linked to antigenic variation, the main instrument of parasite virulence. The expression of a second variant surface glycoprotein (VSG) leads to transcriptional attenuation of the VSG expression site (ES) and immediate development to tsetse fly infective stumpy parasites. This path is independent of SIF and solely controlled by the transcriptional status of the ES. In pleomorphic trypanosomes varying degrees of ES-attenuation result in phenotypic plasticity. While full ES-attenuation causes irreversible stumpy development, milder attenuation may open a time window for rescuing an unsuccessful antigenic switch, a scenario that so far has not been considered as important for parasite survival.

## Author summary

African trypanosomes escape the mammalian host’s immune system by antigenic variation of their variant surface glycoprotein (VSG) coat. VSGs are expressed from a specialized region in the genome, the expression site (ES), that contains essential expression site associated genes (ESAGs). So far, it was assumed that only successful antigenic switches to an intact expression site are viable. Here we show that unsuccessful VSG switches are not a dead-end, but may rather contribute to the persistence of the trypanosomes at the population level. We have simulated an unsuccessful VSG switch in pleomorphic trypanosomes by expression of a second VSG from a locus without ESAGs. The parasites responded with surprising phenotypic plasticity. All parasites immediately exchanged the surface coat and reduced the abundance of ES-derived transcripts. However, depending on the degree of ES-attenuation, the transgenic trypanosomes either resumed growth, or stopped proliferation. We show that the growth-arrested populations synchronously differentiate to the stumpy life cycle stage and become infective for the tsetse fly. This occurs at low cell densities and in the absence of the quorum sensing factor SIF. Thus, unsuccessful VSG switches are not lethal and cell density-dependent quorum sensing is not the only path to the tsetse fly competence.

## Introduction

Pathogenic bacteria and protozoan parasites often employ a coat of surface molecules to protect themselves from host immune attack. These surface coats are sometimes variable and hence, not only act as a physical shield but have evolved as an efficient camouflage strategy. The surface-exposed proteins are mostly members of large families and are subject to antigenic variation, i.e. they are sporadically exchanged. This allows the persistence of the pathogens in the host, as well as reinfection. The genetic mechanisms underlying antigenic variation differ greatly, ranging from transcriptional changes in *Plasmodium* to duplicative events for example in *Borrelia* or *Neisseria* [1]. An extensively studied model for antigenic variation is the protozoan parasite *Trypanosoma brucei* and the phenomenon was, in fact, first described in trypanosomes [2,3]. The surface coat of trypanosomes consists of millions of identical copies of a variant surface glycoprotein (VSG) [4,5]. The highly immunogenic VSGs cause a rapid host immune response, which is thought to lead to an almost complete elimination of the parasite population. Only parasites that have switched to the expression of an immunologically distinct VSG survive. Thus, at any given time just one VSG out of a repertoire of several hundreds of VSG genes is expressed and dominates the cell surface of the pathogen [6,7]. At all times the parasite has to maintain the shielding function of the coat and hence, the concentration of VSGs on the cell surface. This is not a straightforward task as the VSG coat is continuously endocytosed and recycled with unprecedented kinetics [8]. Consequently, VSGs are constantly produced in large quantities. Uniquely, this high level expression of VSG is driven by RNA-polymerase I [9].

*T*. *brucei* exploits both genetic and epigenetic mechanisms for antigenic variation [10,11]. Allelic exclusion, which may be achieved by epigenetic modifications [12,13], ensures that only one VSG gene is expressed from one of 15 telomeric expression sites (ES) [14]. The open chromatin structure of the active ES is thought to facilitate its transcription by RNA polymerase I in a distinct extranucleolar compartment termed the expression site body (ESB) [15–17]. The large repertoire of silent VSG copies is subject to frequent rearrangements, resulting in the continuous production of new mosaic variants [7,18,6,19]. A VSG switch is recombinational when the actively transcribed VSG gene is replaced by another variant. Besides by gene conversion, antigenic variation can occur by telomere exchange, i.e. by recombinational cross-over of chromosome ends [20,21]. Alternatively, the expressed VSG can be exchanged by transcriptional silencing of the active ES and activation of another, previously non-transcribed ES [22]. This so-called ‘*in situ* switch’ does not involve genetic recombination but possibly epigenetic modifications [13]. Since VSG ESs are polycistronic transcription units, an *in situ* switch also silences the expression site associated genes (ESAGs). The number and order of ESAG genes can vary between ESs and not all ESAGs have been functionally characterized [14]. Irrespective of the mode of VSG switching, the *VSG* mRNA levels must be kept rather constant, as down-regulation of *VSG* mRNA rapidly leads to cell cycle arrest followed by parasite death [23]. Therefore, recombinational switches have to be fast, and the activation of a new ES should precede silencing of the old one during a transcriptional switch.

Antigenic variation is the trypanosome’s key-strategy for establishing a persistent infection in the mammalian host. For long-term survival, however, the trypanosomes must also limit the burden they impose on the host, as a constantly high parasitemia would be lethal [24]. Consequently, the parasites have evolved a way of limiting their population size: the proliferating slender forms differentiate to the cell cycle arrested and fly-infective stumpy stage. This developmental stage transition is triggered by a quorum sensing mechanism that involves secretion of the ‘stumpy induction factor’ (SIF) [25,26]. In a cell density-dependent manner SIF is thought to accumulate in the bloodstream, and once a threshold is reached, the irreversible transition from the slender to the stumpy bloodstream stage is initiated [27,28]. In this way, the trypanosomes not only regulate their population size, but also promote vector transmission, as only stumpy bloodstream form parasites are thought to establish an infection in the tsetse fly [29]. During stumpy development the protein expression pattern changes as a pre-adaptation for life in the insect [30]. The level of mitochondrial proteins is augmented and the ‘protein associated with differentiation’ (PAD1) is exposed on the surface of stumpy parasites [31,32]. Microscopically, the parasites adopt the eponymous stout appearance, the free flagellum shortens and the mitochondrion elaborates [29,33].

In a previous study we discovered a connection between VSG switching and developmental competence [34]. We simulated the initiation of an *in situ* switch by inducible overexpression of an ectopic VSG. This caused attenuation of the complete VSG ES and growth retardation. The ES-attenuation was dependent on histone H3 methylation, because in the absence of histone methyltransferase DOT1B the phenotype was not detectable. As the growth retardation was accompanied by signs of developmental competence, we hypothesized that attenuation of the active ES might trigger stumpy development.

Thus, in the present work we focused on the question whether, apart from SIF-mediated stumpy formation, there exists a second mechanism that induces stumpy differentiation. Here, we show that SIF is indeed not required for stumpy stage transition and that there is an alternative path, which is controlled by the VSG ES. We propose that the ES represents a switch that interfaces two aspects of parasite persistence: the survival in the host through antigenic variation and the vector transmissibility through stumpy stage development.

## Results

### VSG silencing can be uncoupled from ES silencing

Previous data raised the question whether ectopic VSG overexpression-induced ES-attenuation could cause stumpy differentiation [34]. This is an important point as it would imply that besides the stumpy induction factor SIF, there exists a density-independent trigger for differentiation to the stumpy life cycle stage. This possibility, however, could not be adequately addressed with monomorphic culture forms of *T*. *brucei*, as they have lost the ability to differentiate from the proliferative long slender to the cell cycle arrested short stumpy stage. Only pleomorphic parasites possess full developmental competence and are suitable for analyses of trypanosome differentiation [35,36]. Therefore, we have now exclusively used the pleomorphic trypanosome strain EATRO 1125 (serodeme AnTat1.1) to test whether ectopic VSG overexpression can induce stumpy formation.

**Fig 1:**
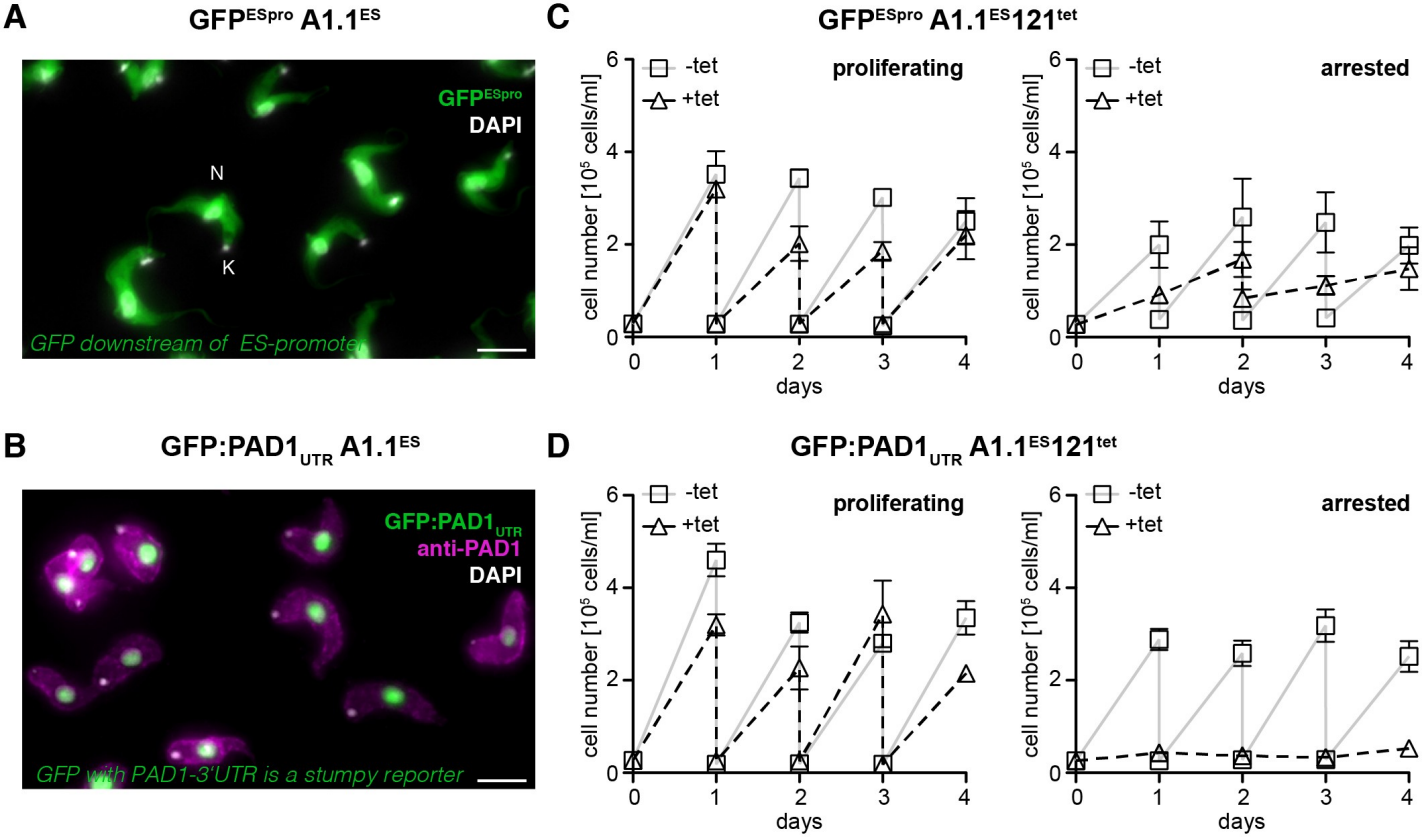
Overexpression of an ectopic VSG causes distinct growth phenotypes. (A) A reporter cell line with a GFP open reading frame integrated into the promotor region of the active AnTat1.1 ES (GFP^ESpro^A1.1^ES^) was generated. The resulting trypanosome clones displayed a homogenous cytoplasmic GFP-signal (green). Nuclear (N) and mitochondrial DNA (K) were stained with DAPI (white). Scale bar: 5 μm. (B) A stumpy reporter cell line with a GFP:PAD1_UTR_ construct integrated into the tubulin locus was generated (GFP:PAD1_UTR_A1.1^ES^). The transgenic trypanosomes were adjusted to 5x 10^5^ cells/ml and cultivated for two days without dilution. As a consequence of cell density induced quorum sensing, the reporter cell line expressed the stumpy GFP-reporter in the nucleus (green) and the endogenous surface protein PAD1 on the plasma membrane (anti-PAD1 antibody; magenta). Scale bar: 5 μm. (C) Transfection of the ES-promoter reporter line (GFP^ESpro^A1.1^ES^) with the inducible VSG 121 overexpression construct (121^tet^) yielded the GFP^ESpro^A1.1^ES^121^tet^ cell lines, while (D) transfection of the stumpy reporter cell line with 121^tet^ yielded the GFP:PAD1_UTR_A1.1^ES^121^tet^ cell lines. (C, D) After induction of VSG121 overexpression clonal populations of both reporter cell lines revealed different growth phenotypes. The trypanosomes either continued (proliferating) or ceased growth (arrested). Only the arrested clones expressed the GFP:PAD1_UTR_ stumpy reporter (S8 Fig). Representative growth curves of tetracycline-induced (triangles) and non-induced cells (squares) of proliferating and growth arrested clones are shown. Each graph represents one clone and the data are means (± SD) of three experiments. Cumulative growth curves, including one of the parental AnTat1.1 cell line as a control, are shown in S2 Fig.

We initially established two reporter cell lines in parasites natively expressing the VSG AnTat1.1 (A1.1). The first cell line was generated to monitor the activity of the VSG ES. A GFP open reading frame was integrated just downstream of the ES-promotor, yielding cell line GFP^ESpro^A1.1^ES^ (Fig 1A, S1 Fig). The second trypanosome line was produced to observe a gain in developmental competence. The fluorescent stumpy stage reporter GFP:PAD1_UTR_ was integrated into the tubulin locus (courtesy of Mark Carrington; [34]). The construct consists of a GFP sequence with a nuclear localization signal, followed by the 3`UTR of the stumpy-specific ‘protein associated with differentiation 1’ (PAD1). A sequence motif in the 3`UTR mediates the early increase of PAD1 transcript abundance during stumpy development [37]. Therefore, the nuclear fluorescence of GFP:PAD1_UTR_ reflects the expression of the cell surface protein PAD1, and hence, is a direct indication for stumpy development (Fig 1B). For ectopic overexpression of VSG 121, a pLew82v4 construct, which inserts into the ribosomal spacer region, was used. The inducible ectopic VSG overexpression is driven by a T7-polymerase under the control of a tetracycline repressor. The construct for ectopic VSG overexpression was transfected into both reporter lines, generating the trypanosome lines GFP^ESpro^A1.1^ES^121^tet^ (Fig 1C) and GFP:PAD1_UTR_A1.1^ES^121^tet^ (Fig 1D).

**Fig 2:**
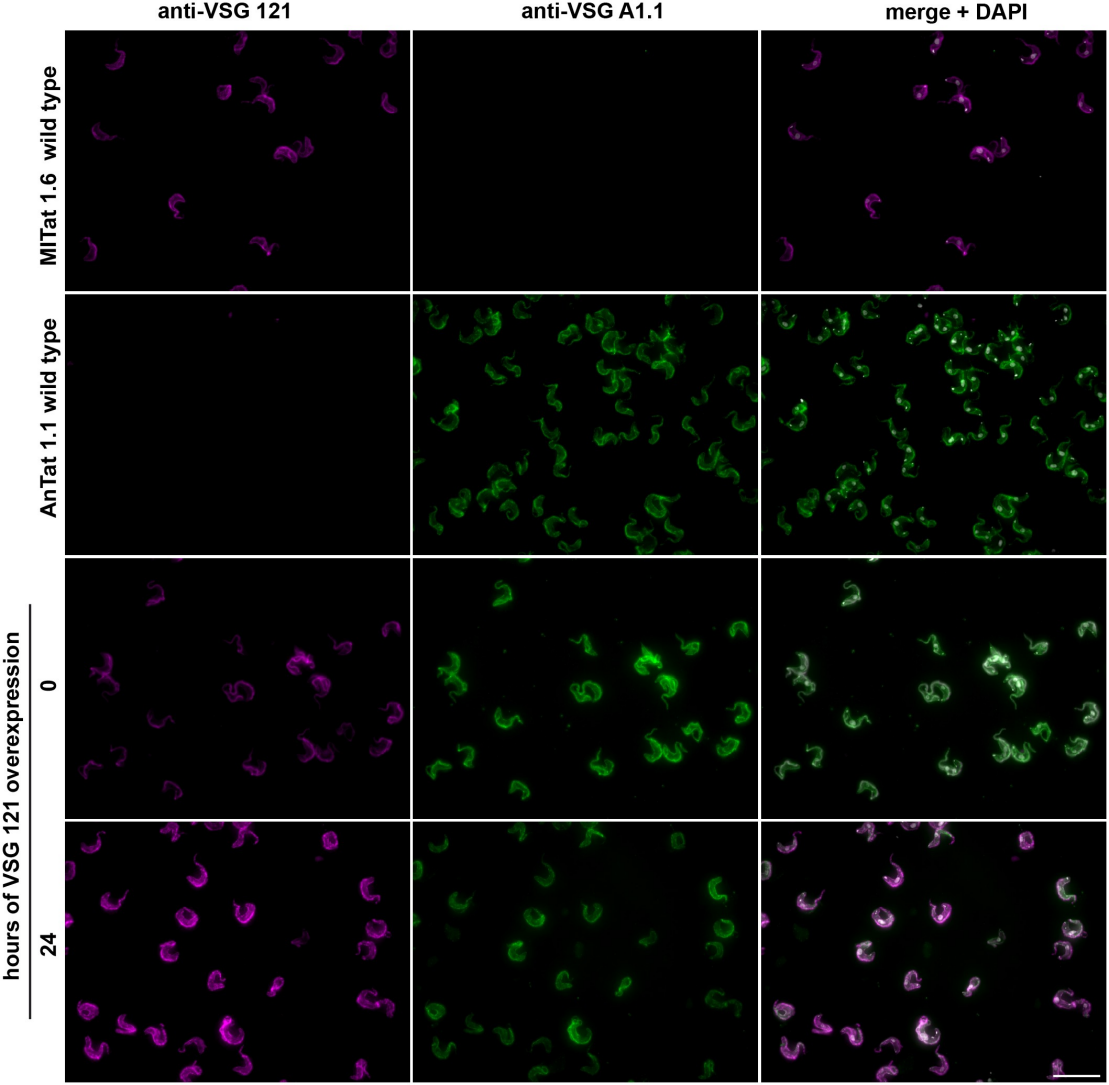
Overexpression of an ectopic VSG causes surface coat exchange. Immunostaining of a proliferating GFP:PAD1_UTR_A1.1^ES^121^tet^ clone using antibodies against the ectopic VSG 121 (magenta, left) and the endogenous VSG A1.1 (green, middle). MITat1.6 wild type cells natively expressing VSG 121 and AnTat1.1 wild type cells natively expressing VSG A1.1 were used as controls for antibody specificity. Non-induced cells (0) and VSG overexpressing parasites induced for 24 hours were analyzed. The merged images are shown in the right panel. DNA stained with DAPI (grey) is displayed in the merged image only. Scale bar: 20 μm. Flow cytometry quantifications of parasites stained with an antibody against the ectopic VSG 121 are presented in S3 Fig.

The ectopic overexpression of VSG 121 yielded clones with different growth phenotypes. In a subset of clones, the parasites continued to grow with only slightly impaired population doubling times (Fig 1C and 1D, proliferating). In other clones the parasites stopped growth after one cell cycle (Fig 1C and 1D, arrested). Irrespective of the cell cycle response, all clones expressed the induced ectopic VSG 121 on the cell surface, as was revealed by immunofluorescence analyses. An example of a proliferating VSG overexpressor is shown in Fig 2 and flow cytometry analysis of the same clone in S3 Fig. The distinct responses of the parasite clones were not due to expression of a specific VSG, but were reproduced with another VSG. The ectopic overexpression of VSG 118 had either no effect on growth or initiated a rapid growth arrest. Irrespective of the growth response, the trypanosomes exchanged their cell surface coat, now presenting VSG 118 on their surface (S4 Fig). Thus, ectopic VSG overexpression mimics an antigenic switch of VSG coats.

**Fig 3:**
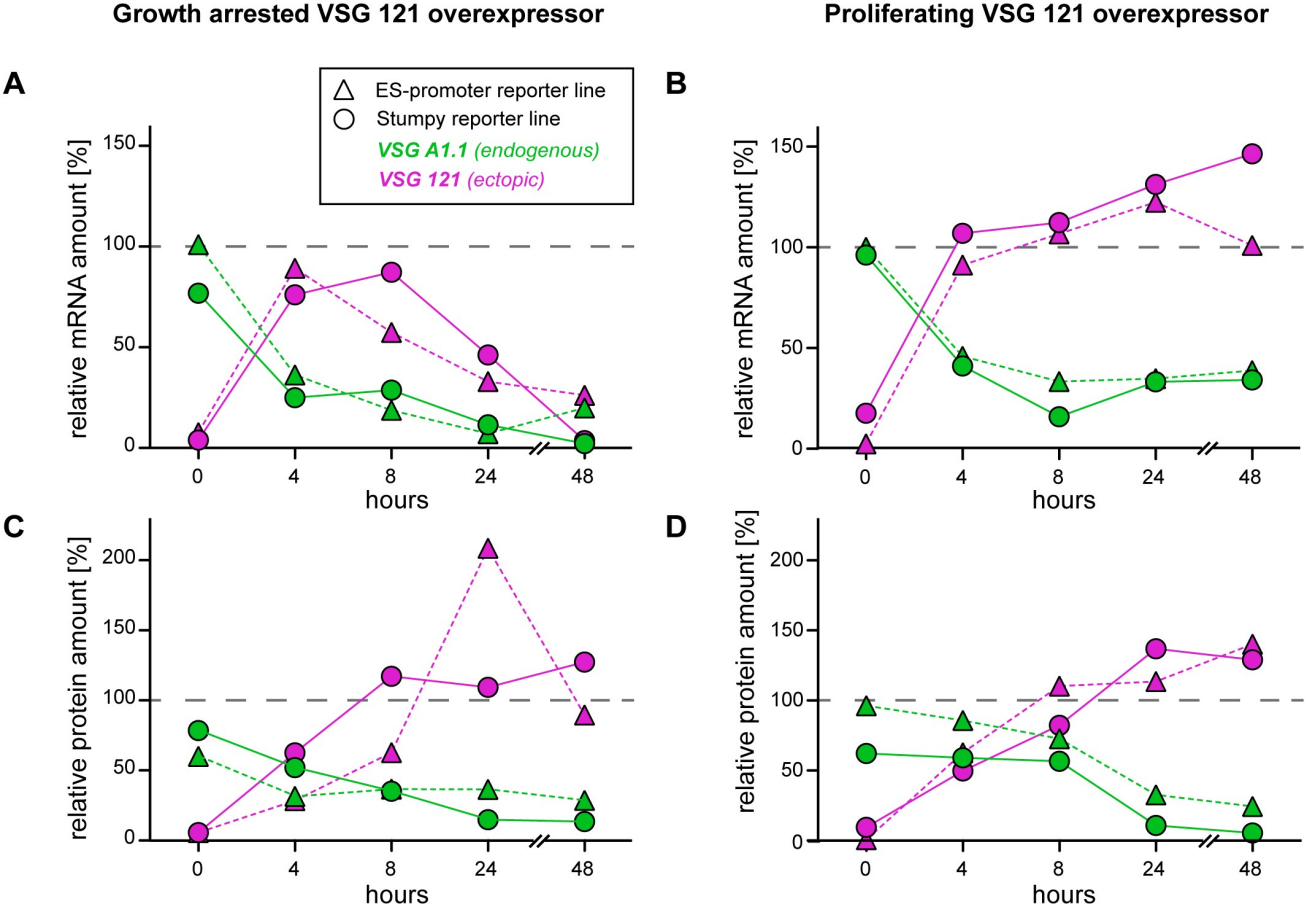
Overexpression of an ectopic VSG causes silencing of the ES-resident VSG. Both (A, B) mRNA and (C, D) protein levels of the endogenous VSG A1.1 (green) and the ectopic VSG 121 (magenta) were monitored in growth arrested (left) and proliferating (right) clones during the course of ectopic VSG overexpression. In all graphs triangles correspond to the ES-promoter cell line (GFP^ESpro^A1.1^ES^121^tet^) and circles to the stumpy reporter cell line (GFP:PAD1_UTR_A1.1^ES^121^tet^). Note that the initial VSG overexpression levels are comparable in arrested and proliferating parasites. For the quantification of (A, B) mRNA levels, total RNA samples were dot-blotted and hybridized with infrared fluorescently labeled probes, specific for *VSG 121* or *VSG A1*.*1*. The data were quantified and normalized to *β-tubulin* mRNA using the Licor Odyssey system. (C, D) VSG protein levels were quantified by dot-blotting 6x 10^5^ cell equivalents. The blots were incubated with an anti-VSG 121 or an anti-VSG A1.1 antibody. A histone H3 antibody was used for normalization. The VSG expression levels are given relative to VSG 121 expression levels of MITat1.6 wild type cells and parental AnTat1.1 cells natively expressing VSG A1.1. The dashed grey line indicates wild type expression levels (100%).

Quantitative Northern blot analyses documented the very fast kinetics of ectopic *VSG 121* mRNA expression and the virtually simultaneous loss of native *VSG A1*.*1* mRNA. In both, proliferating and arrested cells, the induction of ectopic VSG 121 overexpression led to an increase in *VSG 121* mRNA to wild type levels within 4 hours (Fig 3A and 3B). In the same period, the transcripts of the endogenous *VSG A1*.*1* dropped to below 50%. Likewise, within 8 hours of induction, the protein levels of VSG 121 increased to ES-levels in both proliferating and arrested populations (Fig 3C and 3D). The amount of the endogenous VSG A1.1 protein decreased in both cases to about 25% within 24 hours. After 8 hours, the amount of *VSG 121* transcripts started to decrease in growth arrested clones, whereas in proliferating parasites the levels of *VSG 121* mRNA remained constant. The VSG 121 protein was highly expressed in all trypanosome lines. Thus, after 24 hours of induction, the ectopic overexpression of VSG 121 always resulted in an almost complete exchange of VSG coats. Consequently, the different growth phenotypes could not be explained by differences in VSG coat exchange.

**Fig 4:**
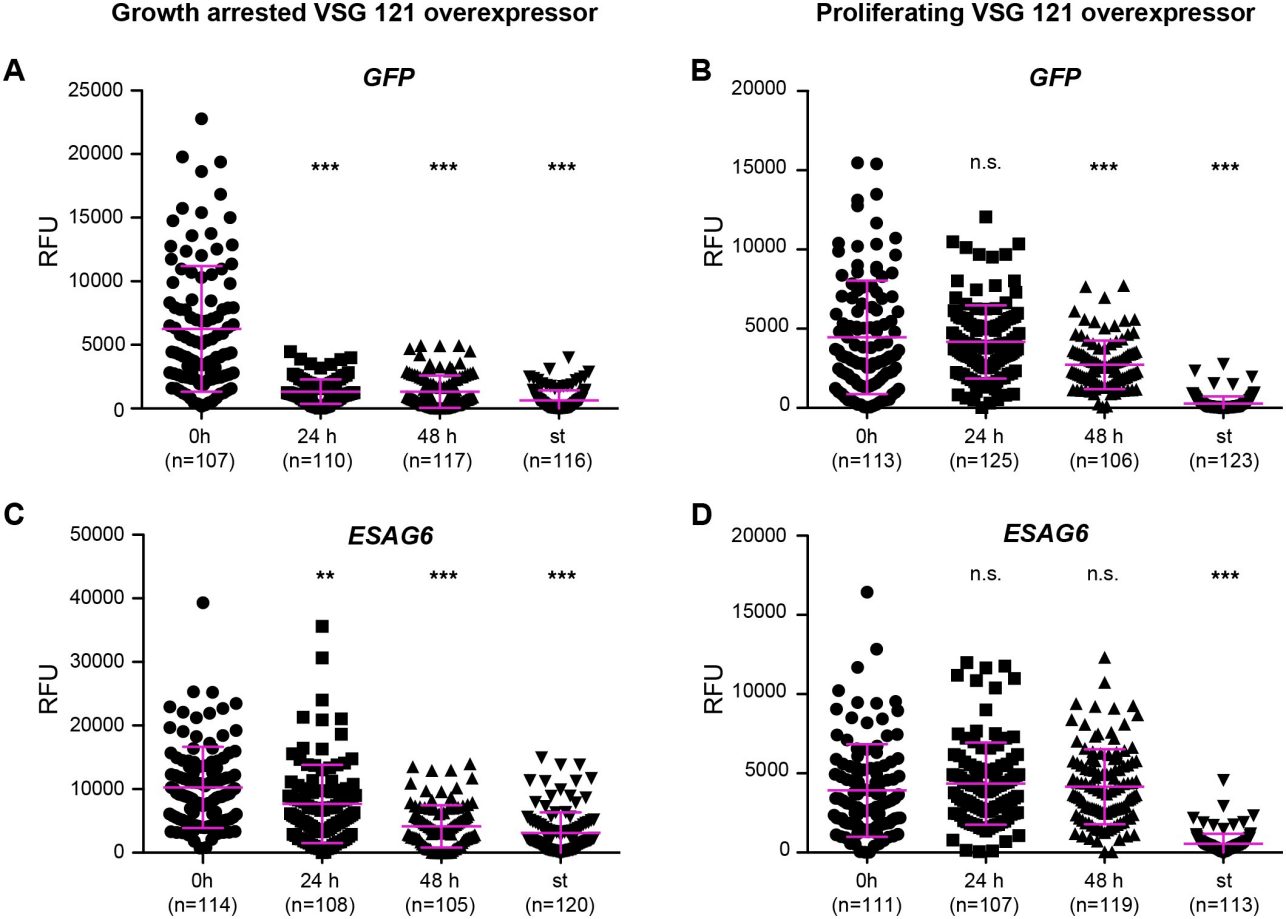
Silencing of the ES-resident VSG is independent of ES-attenuation. The transcriptional status of the active ES of growth arrested (left) and proliferating (right) ectopic VSG 121 overexpressors was monitored. (A, B) The GFP^ESpro^ reporter was used to detect transcripts from the ES promotor region (*GFP*) and (C, D) *ESAG6* was measured as an example for a native ES transcript. All data points reflect measurements on the single cell level using mRNA FISH (Affymetrix). The signal intensity in deconvolved, summed slice projections (100 images, z-step 100 nm) was measured with Image J and is represented as relative fluorescent unit (RFU). Two different induction times (24 and 48 h) were analyzed, and non-induced long slender (0 h) or density-induced short stumpy cells (st) served as controls. Only parasites in G1-phase of the cell cycle were analyzed. The magenta bars are means ± SD (n > 100). Statistical analysis was conducted using an unpaired t-test (not significant (n.s.) p-value > 0.05; ** p-value < 0.01; *** p-value < 0.001).

Therefore, we assessed the transcriptional status of the A1.1 ES to determine if the phenotypes were the consequence of differences in ES-activity. The promotor proximal *GFP* reporter mRNA was quantified using Northern blot analyses (S5 Fig). In growth-arrested trypanosomes, the *GFP* mRNA decreased to less than 50% within 24 hours, when compared to non-induced cells, suggesting that the ES was less active (S5A Fig). In the same period of time, *GFP* mRNA levels in proliferating VSG 121 overexpressors remained above 70% indicating that the ES was more active (S5B Fig). These results were confirmed at the single cell level using quantitative *in situ*-hybridization (Fig 4). In growth arrested cells, the *GFP* mRNA signal dropped by 80% within the first 24 hours, and remained at this low level for two days (Fig 4A). As a control, G1/0 arrested short stumpy trypanosomes (st) were used, because in this life cycle stage the ES is attenuated [38]. In the density-induced stumpy trypanosomes the *GFP* mRNA was down-regulated by 90%, when compared to the long slender stage (0 h) of the same strain. In proliferating clones, the *GFP* mRNA levels remained unaffected for 24 hours, whereas after 48 hours of ectopic VSG 121 overexpression, the mRNA had decreased to 50% compared to slender cells (Fig 4B). This emphasized that proliferating clones also had reduced the ES-activity, however, without consequences for cell growth. Transcript levels, monitored for another endogenous component of the active ES, supported these results. We quantified the transcripts of *ESAG6*, encoding part of the essential trypanosome transferrin receptor, which is present in all ESs [14]. In a growth arrested clone, ectopic VSG overexpression lead to a decrease of *ESAG6* mRNA levels to 75% within 24 hours when compared to the non-induced control (Fig 4C) (unpaired t-test: p-value < 0.01). After 48 hours, the *ESAG6* mRNA had further decreased to 40%, which was comparable to the amount measured in the density-induced stumpy cells (30%). No significant changes in *ESAG6* mRNA levels were detected in the proliferating population within 48 hours of ectopic VSG 121 overexpression (Fig 4D). Thus, the very sensitive single-cell measurements supported the results obtained from Northern analyses.

In summary, our results so far showed that in cells ectopically overexpressing VSG 121, attenuation of the complete ES led to a growth arrest. In contrast, in proliferating clones the endogenous VSG gene was silenced, while other parts of the ES largely retained their transcriptional activity. Importantly, the induced VSG coat exchange was stable over prolonged periods in proliferating clones, as after one month the VSG 121 still dominated the VSG coat in the majority of the cells (S6 Fig). Thus, all these experiments suggest that the VSG and the ES can be silenced independently and that this uncoupling can be stable for many parasite generations. In addition, it confirms that the VSG-coat forming mRNA neither has to be transcribed from a telomeric position nor from the active ES.

### ES-attenuation triggers differentiation to the short stumpy life cycle stage

**Fig 5:**
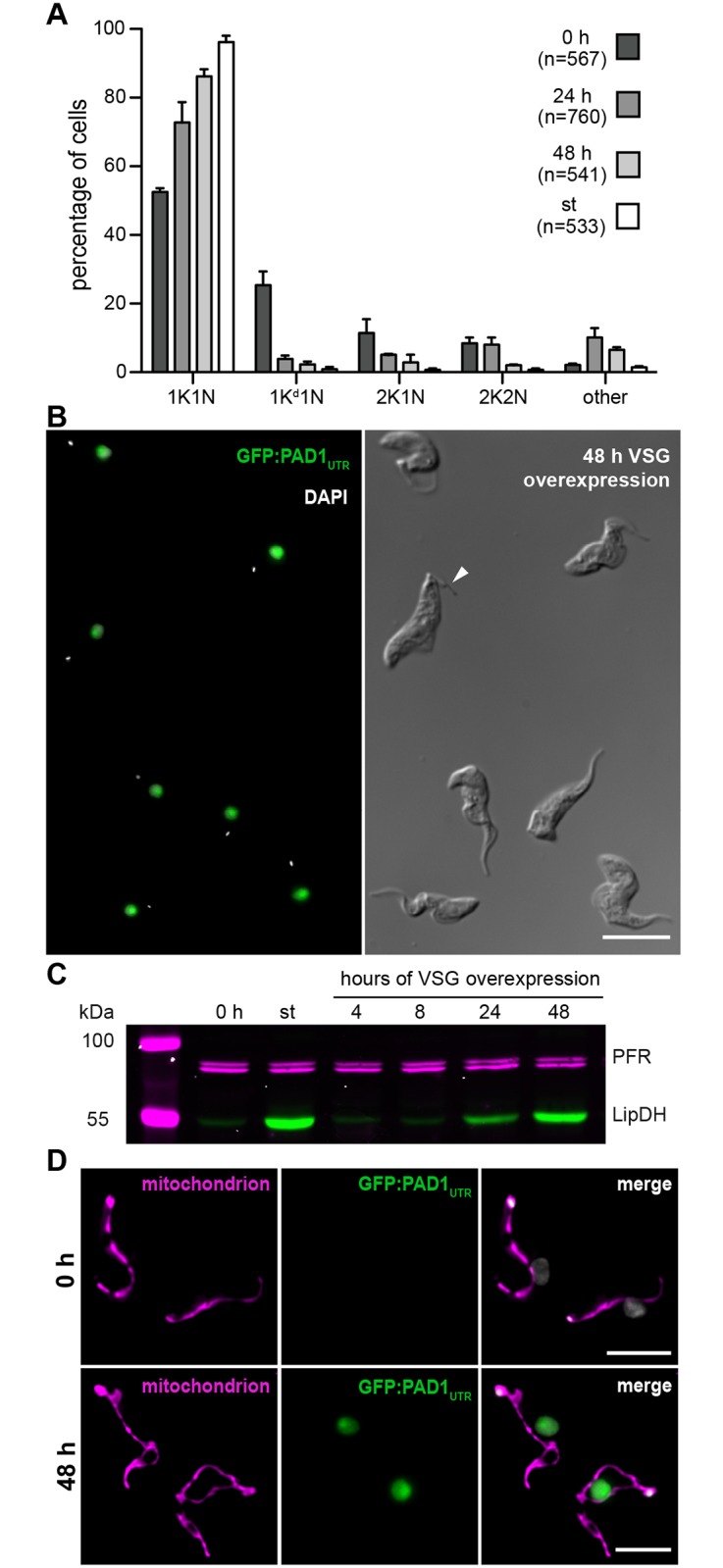
Ectopic VSG overexpression-induced ES-attenuation causes stumpy development. The analyses were conducted with an arrested clone of the GFP:PAD1_UTR_ reporter cell line at densities below 5x 10^5^ cells/ml (except for the density-induced control). (A) The cell cycle position of DAPI-stained trypanosomes was analyzed after 24 and 48 hours of tetracycline induction. Non-induced slender (0 h) and density-induced stumpy cells (st) served as controls. The configuration of the kinetoplast (K) and nucleus (N) was microscopically analyzed. Only cells with a non-dividing kinetoplast and a single nucleus are in the G1-phase (1K1N). Parasites with a dividing kinetoplast (1K^d^1N) are in the mitochondrial S-phase. Trypanosomes with two kinetoplasts (2K1N) are in G2/M-phase, while parasites with two kinetoplasts and nuclei (2K2N) are post mitotic. Parasites with an abnormal K/N configuration were termed "other". Values are given as percentages (± SD) of two experiments (total n > 500). (B) Representative microscopic image of cells ectopically overexpressing VSG 121 for 48 hours. In the left image DAPI (grey) and GFP:PAD1_UTR_ fluorescent signals (green) illustrate the 1K1N cell cycle arrest and the expression of the stumpy stage reporter. The DIC image on the right documents the short stumpy morphology of the trypanosomes. Note the characteristic short free part of the flagellum (white arrowhead). Scale bar: 10 μm. (C) Western blot stained with an antibody against the mitochondrial lipoamide dehydrogenase (LipDH, green), whose expression increases during stumpy development. This shows that LipDH is up-regulated during ectopic VSG overexpression-induced ES-attenuation. Detection of paraflagellar rod (PFR) proteins served as a loading control (magenta). (D) Deconvolved three-channel 3D images of chemically fixed non-induced slender cells (0 h, upper panel) and parasites ectopically overexpressing VSG 121 for 48 hours (48 h, lower panel). The mitochondrion was stained with mitotracker (magenta), the GFP:PAD1_UTR_ reporter is shown in green and DAPI in grey. The upper panels show the shape of typical elongated mitochondria of slender cells, and the lower ones branched stumpy-like mitochondria of ectopic VSG overexpressors (48 h). Scale bars: 5 μm.

Growth arrested ectopic VSG overexpressors had an attenuated ES. To test if the growth phenotype was linked to a specific cell cycle stage, we determined the kinetoplast/nucleus (K/N) configuration (Fig 5A). An accumulation of non-dividing 1K1N cells (G1-phase) was detected in growth arrested clones. Already after 24 hours of ectopic VSG 121 overexpression 20% more 1K1N cells were present in the population compared to non-induced slender cells (0 h), and after 48 hours of induction, 86% of the parasites were in G1. At the same time, the number of dividing cells (1K^d^1N, 2K1N and 2K2N) had decreased. Thus, the parasites were stalled in the G1/0-phase of the cell cycle, very much like the density-induced stumpy control (st), which per definition is G1/0 arrested [39]. In addition, the ectopic VSG overexpressors changed their morphology within 48 hours of induction, now displaying the characteristic shortened flagellum and stout appearance of density-induced short stumpy parasites (Fig 5B). Next, we tested for the presence of the green fluorescent GFP:PAD1_UTR_ reporter, which is exclusively expressed in the stumpy stage [32]. After 24 hours of ectopic VSG overexpression, already 74±4% of all cells expressed the reporter. After 48 hours, 90±9% of the cells displayed a green fluorescent nucleus (S7A Fig), which was comparable to the number of cells expressing the GFP:PAD1_UTR_ reporter in density-induced stumpy parasites of the same cell line (97±3%; S7A Fig). We also analyzed the expression of a second protein that is strongly up-regulated during stumpy development, the mitochondrial lipoamide dehydrogenase (LipDH) [31]. Western blot analyses showed that after 48 hours of ectopic VSG overexpression, LipDH increased 10-fold in growth arrested clones, when compared to non-induced long slender cells (0 h) (Fig 5C). Thus, in ectopic VSG overexpressors, LipDH levels were similar to those of density-induced stumpy parasites. Next, the morphology of the mitochondrion, which is another hallmark for the discrimination of slender and stumpy parasites, was assessed. The organelle grows and branches during stumpy development as a metabolic pre-adaption to the loss of glucose homeostasis, which occurs upon uptake by the transmitting tsetse vector [29,40]. The morphology of the mitochondrion was visualized using Mitotracker in arrested ectopic VSG overexpressors (48 h) and in non-induced slender parasites (0 h) (Fig 5D). As a control for mitochondrial expansion, density-induced short stumpy trypanosomes (st) of the same cell line were used (S7B Fig). As expected, the mitochondria in the slender control trypanosomes had the characteristic slim and elongated shape. After 24 hours of VSG 121 overexpression, 70±9% of the parasites possessed a branched mitochondrion (S7B Fig). After 48 hours of induction, 87±5% of the cells displayed a branched mitochondrion, which compares well to 90±4% in density-induced stumpy trypanosomes. Another, more subtle marker for stumpy differentiation is an increase in the expression of the glycosomal DxDxT class phosphatase PIP39 [41]. This protein is essential for stumpy to procyclic transition, as it is part of the citrate/*cis*-aconitate (CCA) signaling cascade, which promotes procyclic development upon entry of the stumpy parasites into the alimentary system of the tsetse fly [42]. Western blot analysis showed that PIP39 is upregulated in density-induced stumpy parasites, as well as in a growth arrested clone after 24 and 48 hours of ectopic VSG overexpression (S7C Fig). Thus, VSG-induced ES-attenuation initiates growth arrest in G1/0, expression of stumpy marker proteins, mitochondrial re-organization and changes to a stumpy cell morphology. We conclude that ectopic VSG 121 overexpressors with an attenuated ES are indistinguishable from density-induced short stumpy trypanosomes. Therefore, we introduce the term ‘ES-attenuation-induced stumpy trypanosomes’ for such cells.

The stumpy development observed above was not the result of cell stress. As a control, we exposed slender parasites to mild acid conditions (pH of 5.5) for 30 minutes and two hours, as reported by Rolin et al. [43] (S8A Fig). Propidium iodide (PI) staining of the stressed cells showed that 30 minutes of mild acid treatment was sufficient to kill the majority of cells (PI-positive). After 2 hours virtually no living cells (PI-negative) could be detected. Parasites treated for 30 minutes were washed and cultivated further to analyze if the surviving cells would differentiate to the stumpy stage. However, the parasites grew normally and did not arrest in the cell cycle (S8B Fig), which means the surviving cells were slender stage trypanosomes. This was supported by monitoring the GFP:PAD1_UTR_ stumpy reporter 24 and 48 hours after mild acid treatment (S8C Fig). No increase in the number of fluorescent stumpy parasites was detected. Thus, mild acid treatment does not trigger stumpy development in slender parasites.

**Fig 6:**
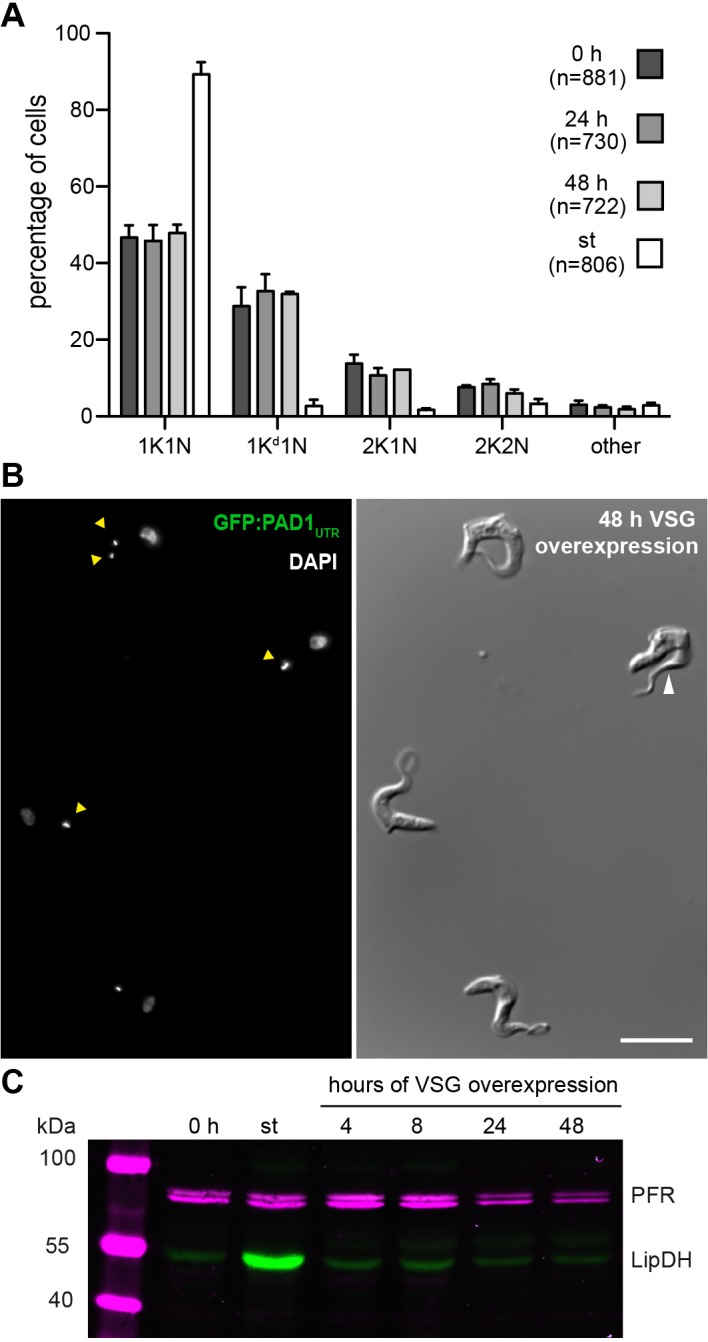
VSG silencing without ES-attenuation is not sufficient to trigger stumpy differentiation. The experiments were conducted with a proliferating clone of the GFP:PAD1_UTR_ reporter cell line at densities below 5x 10^5^ cells/ml (except the density-induced control). (A) The cell cycle position of DAPI-stained trypanosomes was analyzed after 24 and 48 hours of tetracycline induction. Non-induced slender (0 h) and density-induced stumpy cells (st) served as controls. Values are given as percentages (± SD) of two experiments (total n > 700). (B) On the left DAPI staining (grey) illustrates different dividing stages (indicated by yellow arrowheads). The green GFP:PAD1_UTR_ stumpy marker signal is absent. The DIC image on the right illustrates the typical slender morphology of proliferating ectopic VSG overexpressors. Note the characteristic extended free part of the flagellum (white arrowhead). Scale bar: 10 μm. (C) Western blot stained with an antibody against the mitochondrial lipoamide dehydrogenase (LipDH, green), whose expression increases during stumpy development. This reveals the uniformly low LipDH expression in proliferating ectopic VSG overexpressors during the time course of induction. Detection of paraflagellar rod (PFR) proteins served as a loading control (magenta).

Proliferating ectopic VSG 121 overexpressors that had exchanged the VSG surface coat, but maintained an ES-activity of above 50%, did not show any alterations in the cell cycle (Fig 6A). Following induction of overexpression the parasites retained their slender morphology (Fig 6B) and did not express the GFP:PAD1_UTR_ reporter (Fig 6B and S9A Fig). LipDH expression remained at the same level as in non-induced slender cells (Fig 6C) and no mitochondrial restructuring could be observed (S9B Fig). This all suggested that ES-attenuation is required to induce differentiation, whereas silencing of the ES-resident VSG alone does not lead to stumpy development. However, at this point we had not formally excluded that the proliferating clones simply were refractory to stumpy induction. To test this, non-induced parasites were grown to high densities to induce stumpy development. In this control we observed a G1/0 cell cycle arrest in more than 90% of the parasites (st in Fig 6A), expression of the GFP:PAD1_UTR_ reporter in a high proportion of the cells (st in S9A Fig) and an increase in the LipDH levels (st in Fig 6C).

**Fig 7:**
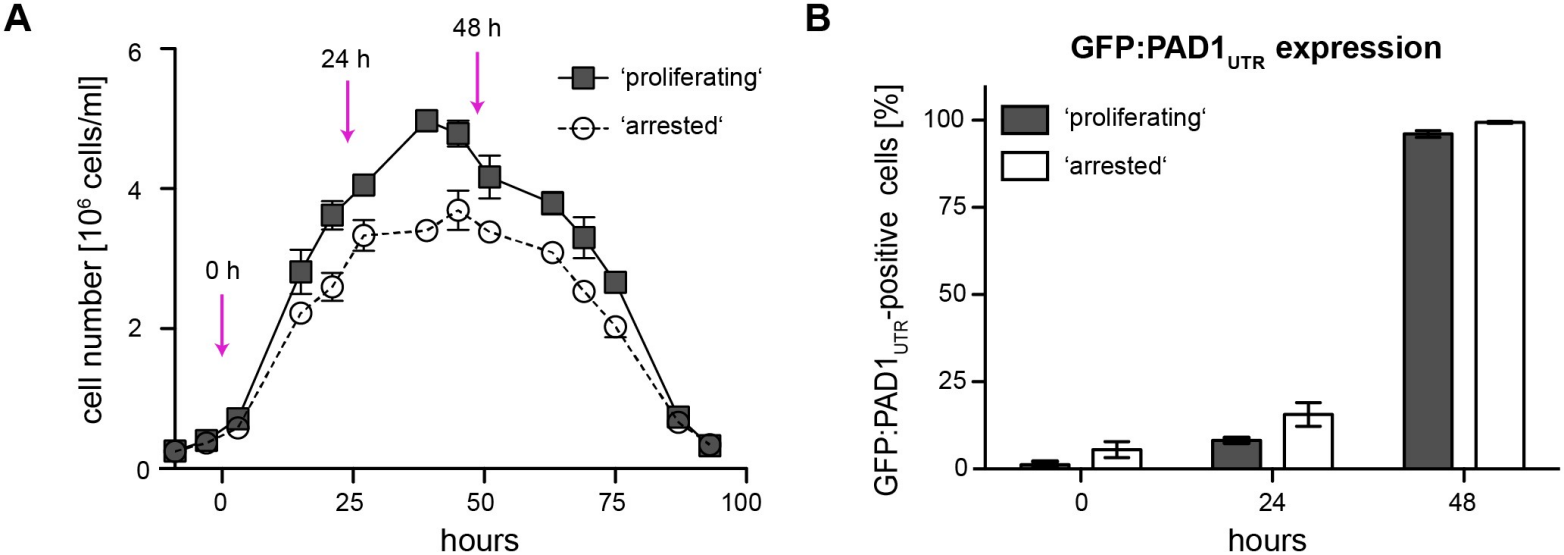
Proliferating and arrested clones respond to SIF with development to the stumpy stage. Slender parasites of a proliferating and an arrested clone of the GFP:PAD1_UTR_ reporter cell line were cultivated without dilution. This allowed the accumulation of the parasite secreted stumpy inducing factor (SIF). This was done in the absence of tetracycline, i.e. without VSG overexpression. Therefore, we here refer to potentially proliferating ('proliferating') and potentially arrested ('arrested'). (A) Representative growth curves of a ‘proliferating’ (squares) and an ‘arrested’ (circles) clone. The arrows indicate the time points at which the number of GFP:PAD1_UTR_-positive cells was determined (see B). Control growth curves for the parental GFP:PAD1_UTR_ reporter cell line are shown in S10 Fig. Data are means (± SD) of three experiments. (B) Quantification of cells expressing the GFP:PAD1_UTR_ reporter. The expression was monitored at cell densities of 5x 10^5^ cells/ml (0 h), as well as after 24 hours and 48 hours of growth. Values are percentages (± SD) of three experiments (total n > 600 cells).

In the next step, parasites were cultivated without dilution in the absence of tetracycline to directly compare the response to SIF of slender populations of proliferating and arrested clones. Population growth was recorded for 96 hours, and the number of GFP:PAD1_UTR_-positive cells indicated SIF-induced stumpy development. As all cells in this experiment were grown in the absence of tetracycline, i.e. without VSG overexpression, they were termed potentially proliferating ('proliferating') and potentially arrested ('arrested'). The ‘proliferating’ clone reached higher cell densities during SIF-induced stumpy development than the ‘arrested’ clone (Fig 7A). Nevertheless, in both cases the parasite populations synchronously differentiated to the stumpy stage, with more than 90% of the cells expressing the stumpy reporter within 48 hours (Fig 7B). Thus, non-induced slender cells of 'proliferating' as well as 'arrested' clones were fully competent for stumpy development. Thus, attenuation of the ES is required for the induction of stumpy stage transition in the ectopic VSG overexpressors. Silencing of the telomeric VSG alone, however, is not sufficient to induce developmental transition.

### ES-attenuation-induced stumpy trypanosomes passage through the tsetse fly

**Fig 8:**
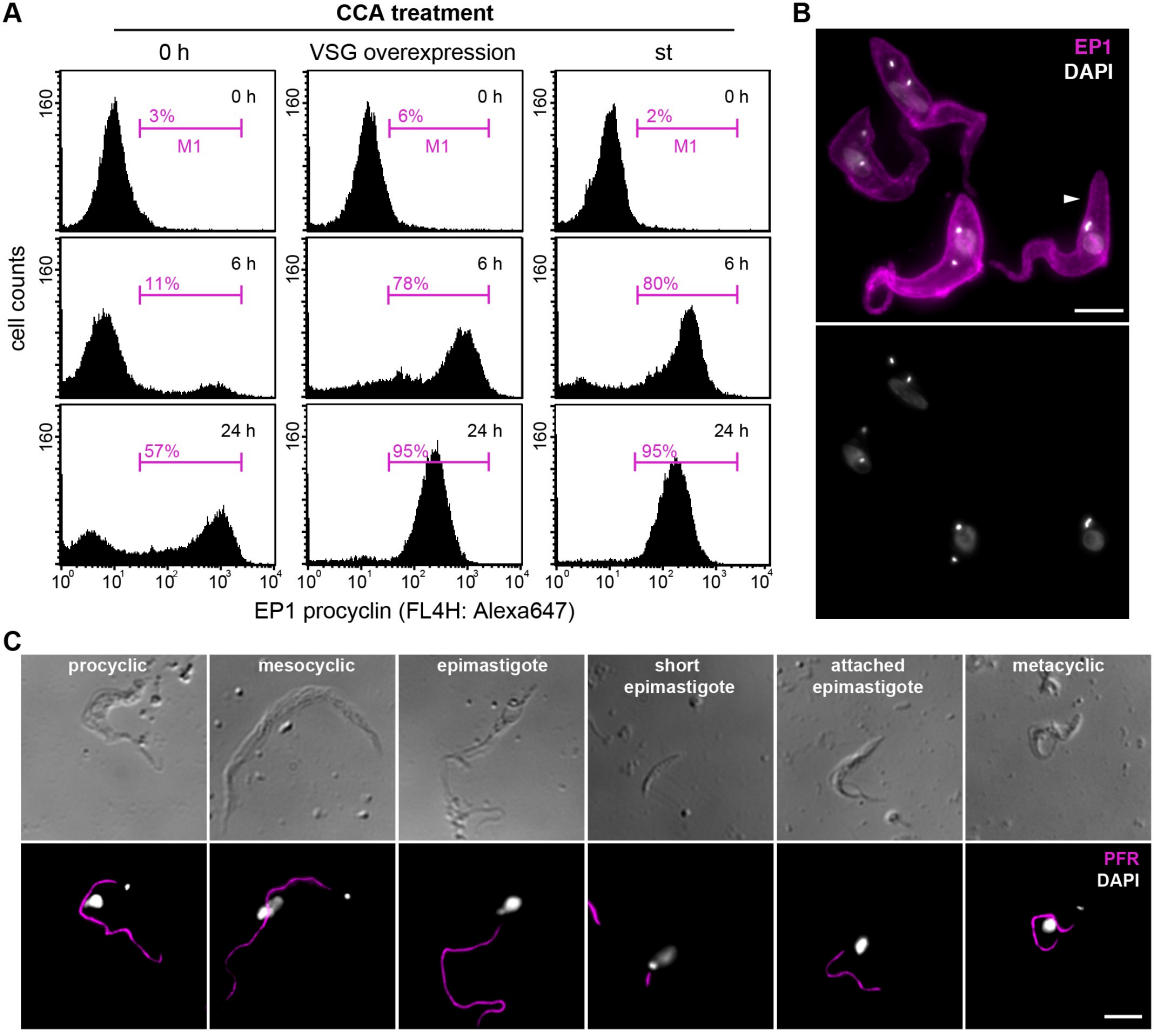
ES-attenuation-induced stumpy trypanosomes possess full developmental competence for tsetse transmission. (A) Flow cytometric analysis of the expression of the procyclic surface protein EP1. ES-attenuation-induced stumpy cells ectopically overexpressing VSG 121 for 48 hours were treated with CCA (3mM *cis*-aconitate and 3mM citrate at 27°C). Non-induced long slender cells (0 h) served as a negative and density-induced stumpy cells (st) as a positive control. After 0, 6 and 24 hours of CCA treatment, the trypanosomes were chemically fixed and immunostained for the detection of EP1. Cells in the M1 region of the plots are EP1-positive. (B) Representative immunofluorescence image of ES-attenuation-induced stumpy cells after 24 hours of CCA treatment. EP1 (magenta) is uniformly distributed on the surface, and the parasites show the characteristic shape of procyclic trypanosomes. Note the elongated posterior pole of the cells (white arrowhead). DAPI staining (grey) confirms the rearrangement of kinetoplast/nucleus and the re-entry into the cell cycle. Scale bar: 5 μm. (C) ES-attenuation-induced stumpy trypanosomes can passage through the tsetse fly. Flies were dissected at the earliest 50 days after infection. Typical parasite stages were present in different infected organs as illustrated by the shape of the cells (DIC images, upper panel). Staining of the flagellum with an antibody against PFR (magenta) shows the characteristic changes in flagellar length. DAPI staining (grey) illustrates the repositioning of kinetoplast and nucleus during developmental progression (lower panel). Scale bar: 5 μm.

Stumpy trypanosomes are thought to be the only bloodstream stage that can infect the tsetse fly [29]. The development of stumpy cells to the procyclic insect stage is accompanied by an early loss of cell surface VSG, which is replaced by an invariant EP-procyclin coat [44,45]. The stumpy-to-procyclic transition can be enforced *in vitro* by cold-shock and treatment with citrate and *cis*-aconitate (CCA) [35,46,47]. Therefore, we challenged growth arrested ectopic VSG 121 overexpressors that displayed stumpy morphology (ES-attenuation-induced stumpy cells) with CCA for 0, 6 and 24 hours at 27°C, followed by immunodetection of EP1 (Fig 8A and 8B). Non-induced long slender cells (0 h) served as a negative control, and density-induced stumpy parasites (st) were used as a positive control. Flow cytometry showed that upon CCA-treatment, both ES-attenuation-induced and density-induced stumpy cells replaced the VSG coat with EP1 (Fig 8A). Within 6 hours, 78% of the ES-attenuation-induced and 80% of the density-induced stumpy parasites were EP1 positive. In contrast, only 11% of the slender forms showed EP1 expression after 6 hours of CCA-treatment. The remarkably similar kinetics of EP1 expression suggested that ES-attenuation-induced and density-triggered stumpy trypanosomes possess the same developmental competence, at least *in vitro*. During procyclic development the parasites elongate at the posterior pole and the kinetoplast is repositioned towards the vicinity of the nucleus [48]. After 6 hours of CCA-treatment, no morphological changes were observed in ES-attenuation-induced stumpy cells. After 24 hours of CCA treatment, however, the distance between kinetoplast and nucleus had shortened from 4.81±0.6 μm to 2.77±0.9 μm. Within the same period, the distance between the posterior cell pole and the kinetoplast almost tripled from 1.45±0.42 μm to 4.13±1.39 μm. Thus, the parasites adopted the characteristic elongated shape of procyclic cells, and the kinetoplast was repositioned towards the nucleus (Fig 8B). Importantly, 33% of the parasites were in the 2K1N or 2K2N cell cycle phase and thus, the trypanosomes had resumed growth as procyclic forms. Hence, the trypanosomes synchronously responded to CCA-treatment with EP1 expression on the surface, loss of the VSG surface coat and morphological alterations that are characteristic for development to the procyclic insect stage. In the next step, we tested if ES-attenuation-induced stumpy parasites would be able to initiate and complete the complex passage through the tsetse vector. For this, ES-attenuation-induced stumpy trypanosomes (2x 10^6^ cells/ml) were included in the blood meal of 50 tsetse flies. Control flies were fed with the same number of density-induced stumpy parasites of the parental GFP:PAD1_UTR_ cell line. After >50 days of infection, the alimentary tract of the flies was dissected and examined for the presence of trypanosomes. Parasites were found in the salivary glands of 6.4% of flies infected with ES-attenuation-induced stumpy parasites (n = 47), whereas in the control experiment density-induced stumpy parasites completed the infection in 22.7% of flies (n = 44). Hence, independent of the differentiation trigger, the stumpy trypanosomes were able to passage through the insect. Fluorescence microscopy was used to probe for the characteristic trypanosome stages in the alimentary system of tsetse flies [49,50]. An antibody against the paraflagellar rod (PFR) visualized the length and location of the flagellum, and the DNA was stained with DAPI to analyze the configuration of kinetoplast and nucleus. All developmental stages of trypanosomes were present in the flies (Fig 8C). Not only procyclic midgut parasites were found, but also mesocyclic, epimastigote and metacyclic stages. Thus, ES-attenuation-induced stumpy trypanosomes are not only able to establish an infection in tsetse flies but can also successfully complete the tsetse passage by developing the mammal-infective metacyclic stage in the salivary glands of the insect.

### Slowly growing trypanosomes that are not fully arrested can escape ES-attenuation induced stumpy development

**Fig 9:**
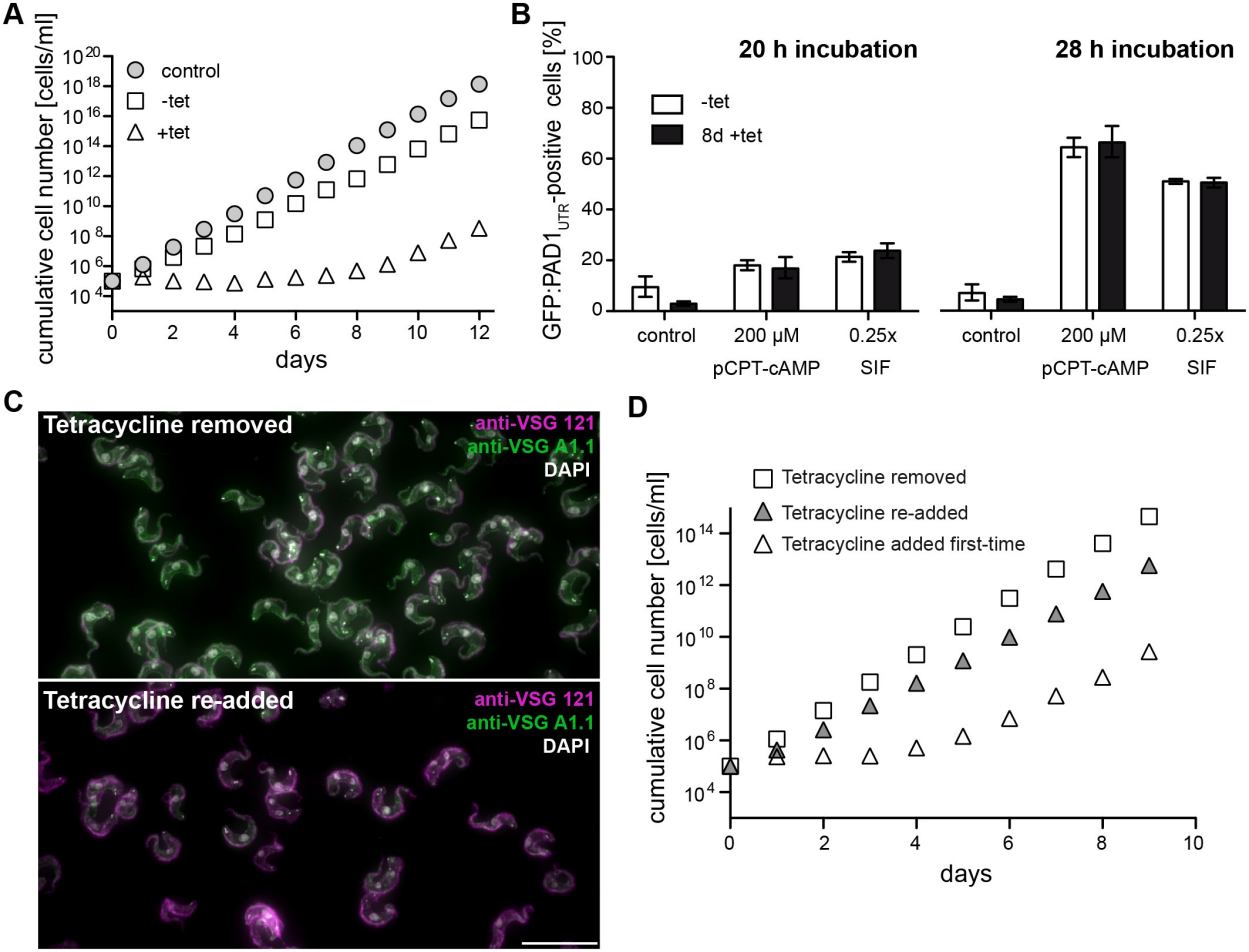
Outgrowing ectopic VSG 121 overexpressors are fully competent for stumpy differentiation and retain the tetracyclin-inducible gene expression system. The analyses were done with a growth arrested clone of the GFP:PAD1_UTR_ cell line that had resumed growth following tetracycline induction. (A) Representative cumulative growth curves of tetracycline-induced (triangles) and non-induced (squares) cells. The parental AnTat1.1 cell line (circles) served as a growth control. Data are means (± SD) of two experiments. Due to the small standard deviation the error bars are not visible. (B) After 8 days of induction, ectopic VSG overexpressors that had resumed growth (8 d tet) and non-induced cells (-tet) were treated with the stumpy-differentiation triggers pCPT-cAMP (200 μM) and SIF (0.25x). Control cells did not receive either compound (control). The number of GFP:PAD1_UTR_ positive cells was microscopically determined after 20 and 28 hours of treatment. Values are percentages (± SD) of experiments performed in triplicate (total n > 600 cells). (C, D) Ectopic VSG overexpression is re-inducible, but growth arrest is not. After 48 hours of ectopic VSG overexpression tetracycline was removed. The parasites were further cultivated without tetracycline for one week before re-addition of the antibiotic. (C) Trypanosomes cultivated without tetracycline for one week did not express the ectopic VSG 121 (magenta), but the ES-resident VSG A1.1 (green). When tetracycline was re-added, the parasites again expressed the ectopic VSG 121 and suppressed VSG A1.1. DNA was stained with DAPI (grey). Scale bar: 20 μm. (D) Cumulative growth curves of trypanosomes after removal (white squares) and re-addition of tetracycline (grey triangles). Tetracycline was removed 48 hours post-induction, and the cells were cultivated for 7 days in the absence of the drug. Then tetracycline was re-added (grey triangles) or not (squares) and population growth was determined. As a control, parasites of the same clone were induced with tetracycline for the first time (white triangles). Data are means (± SD) of three experiments. Due to the small standard deviation the error bars are not visible. For visualisation of the actual cell densities and the standard deviation, the data are presented in S11 Fig as non-cumulative growth curves.

Trypanosome development to the stumpy stage occurs in response to a cell density-dependent quorum sensing mechanism [25]. The parasites continuously secrete SIF, the as yet elusive stumpy induction factor. SIF is thought to accumulate in the host with rising parasitemia and to induce stumpy transition once a concentration threshold is reached [26]. In cell culture, this requires parasite densities of over 10^6^ cells/ml [51]. We postulate that ectopic VSG overexpression-induced ES-attenuation leads to stumpy development in a non-density dependent and, hence, SIF-independent manner. As stumpy cells are irreversibly arrested in the cell cycle and have a lifespan of 2–3 days [52], cell death should become apparent at day 4 post-induction of ectopic VSG overexpression. In fact, we did observe cell death, however, all populations resumed growth at later time points (Fig 9A, S11 Fig). The timing of outgrowth varied and occurred between days 4 and 8 of induction. Several possible mechanisms would explain the outgrowth of the ES-attenuation-induced stumpy parasites: (i) a subpopulation of ES-attenuated parasites is refractory or less sensitive to stumpy differentiation; (ii) the complete A1.1 ES has been re-activated; (iii) a defect in the overexpression system has occurred, e.g. by mutation of the T7 polymerase or promoter; or (iv) a minority of parasites does not attenuate the ES completely and hence, escapes stumpy formation. To test the first possibility, a growing population of trypanosomes that appeared after 8 days of ES-attenuation-induced cell cycle arrest was treated with the stumpy induction factor (SIF), or the downstream signal analogue pCPT-cAMP [26]. If the parasites were refractory to differentiation, they should not respond to these differentiation signals by expression of the GFP:PAD1_UTR_ stumpy reporter. However, both SIF and pCPT-cAMP triggered synchronous differentiation to the stumpy stage with kinetics that were identical to those measured for non-induced parasites (Fig 9B). Explanation (ii) was excluded by immunofluorescence analyses, showing that the outgrowing parasites, even after 28 days, still expressed the ectopic VSG 121 on their cell surface, and thus, had not re-activated the complete A1.1 ES (S12 Fig). The same experiment also precluded (iii) as expression of the ectopic VSG 121 would not be inducible any more if a mutation in the T7 polymerase or promoter had occurred. This was further supported by the finding that the ectopic VSG overexpression system was re-inducible: tetracycline was removed after 48 hours of ectopic VSG 121 overexpression, and the parasites were cultivated for one week without tetracycline. At day 7, the cells had resumed growth and expressed the endogenous VSG A1.1 coat (Fig 9C, top). Then tetracycline was again added to the culture, and within 24 hours, the trypanosomes had once more exchanged their VSG coat, now again predominantly presenting the ectopic VSG 121 on the surface (Fig 9C, bottom). Thus, the outgrowing cells were (i) neither refractory to SIF action, (ii) nor had they re-activated the endogenous VSG A1.1 ES. They were (iii) also not the product of a deficient gene expression system. Interestingly, no growth arrest could be observed when tetracycline was re-added in order to re-induce overexpression of ectopic VSG (Fig 9D, S11B Fig). This means that the outgrowing population was based on parasites that had escaped ES-attenuation-induced stumpy development. The late onset of outgrowth shown in Fig 9A (S11A Fig) excluded that the starting population already contained cells that did not respond to ectopic VSG overexpression with ES-attenuation and subsequent growth retardation. As a fact, short stumpy parasites are cell cycle arrested and can only be rescued by developmental progression to the procyclic insect stage [39,44]. We postulate that this is also true for ES-attenuation-induced stumpy cells. Thus, the late appearing dividing trypanosomes must have escaped ES-attenuation-induced stumpy formation. To estimate the number of escapers, we used serial dilutions. ES-attenuation was induced by ectopic VSG overexpression and the trypanosomes were immediately diluted into 96-well plates, each well containing either 5, 50, 500 or 5,000 cells. As a control for the outgrowing cells, non-induced long slender parasites of the same cell line were used. When 5 cells were seeded per one well, growth resumed in 90% of the control wells, while no growth was observed with induced cells. However, when 50 or 500 induced parasites were seeded per well, cells grew in 1 and 4% of all wells, respectively. Correspondingly, growth was apparent in 40% of wells, which had been seeded with 5,000 induced cells. Assuming that the outgrowing population in one well can originate from just a single cell, at least 1 in 10,000 trypanosomes must have escaped ES-attenuation-induced stumpy formation. However, those cells also most likely had attenuated the ES prior to regaining proliferative capacity. We suggest that all cells in a clonal population respond to ectopic VSG overexpression with ES-attenuation, however, the ES-activity has to fall below a critical threshold in order to drive the cell cycle into the irreversible G1/0 state. In a few parasites, the ES does not reach this critical level. In these cells the native VSG A1.1 is completely silenced, while the A1.1 ES could still provide sufficient ESAG transcripts to support slowed growth. The ectopic VSG 121 continues to be expressed by T7 polymerase. The cells neither effectively proliferate nor do they differentiate into the cell cycle-arrested stumpy stage. They could rather linger in a prolonged G1-phase. This dormancy is an unstable state, which is either drifting towards ES shut-down and subsequent stumpy formation, or it is rescinded by re-activating the ES to permissive levels for re-entry into the cell cycle. In the latter case, the parasites would appear as normally growing long slender trypanosomes, ectopically expressing a VSG 121 surface coat. This was the case for the cells that grew out in the above experiment.

### ES-attenuation induces SIF-independent stumpy development

**Fig 10:**
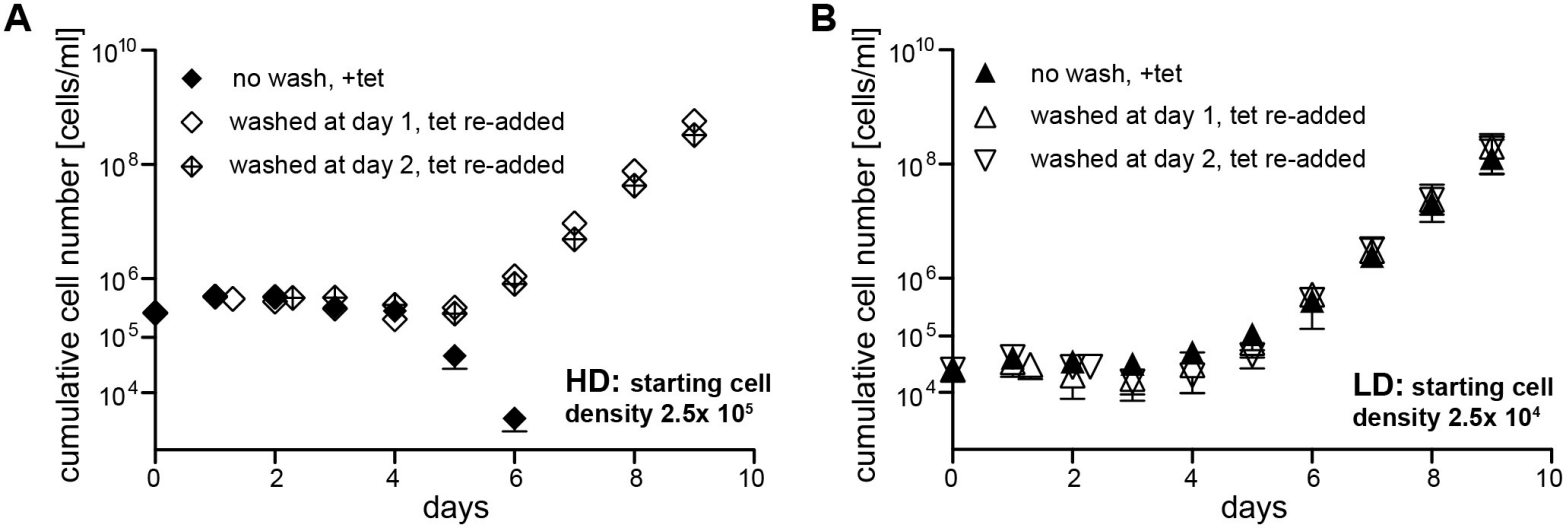
ES-attenuation induces stumpy development independent of cell density and SIF. The possible effects of SIF on a growth arrested clone of the GFP:PAD_UTR_ cell line were determined. Since SIF accumulates in the cell culture medium and induces stumpy development in a cell density-dependent manner, trypanosomes were induced for ectopic VSG overexpression at two different starting cell densities: (A) High parasite density (HD, 2.5x 10^5^ cells/ml) and B) low parasite density (LD, 2.5x 10^4^ cells/ml). The parasites were cultivated without dilution to allow the accumulation of SIF. Cumulative growth curves were recorded (+tet) for 9 days to analyze the possible impact of SIF on the growth arrested ectopic VSG overexpressor. To determine at which time point the ES-attenuation escapers could resume growth, the culture medium was exchanged after either 1 or 2 days of induction by washing (washed at day 1 +tet or 2 +tet). Induction was maintained by re-addition of tetracycline, and cultures were diluted again once they resumed growth. Data are means (± SD) of three experiments. Due to the small standard deviation the error bars are not visible. For visualisation of the actual cell densities and standard deviations non-cumulative growth curves, including non-induced cells and the parental AnTat1.1 cell line as controls, are shown in S13 Fig.

We have shown that ES-attenuation can cause stumpy development and thus, represents a direct differentiation trigger. To further support this finding, we induced ectopic VSG 121 overexpression, and hence ES-attenuation, in a potentially arrested clone at cell densities of 2.5x 10^5^ cells/ml (high density, HD) and 2.5x 10^4^ cells/ml (low density, LD) (Fig 10A and 10B; S13A and S13B Fig). Irrespective of the starting cell densities, the parasites only divided once after tetracycline addition. Thus, for the first four days, the cell numbers never exceeded 5x 10^5^ cells/ml in HD cultures, and 5x 10^4^ cells/ml in LD cultures. At these densities, SIF is initially not present in a sufficient amount for triggering density-induced stumpy development. This excludes that the immediate cell cycle arrest was SIF-driven. The possible action of SIF became evident only at later time points, and only when the HD parasites were incubated without an exchange of the culture medium, which allowed SIF to accumulate in the culture (Fig 10A, no wash; S13A Fig). As the whole population is dying the accumulating SIF could have triggered stumpy formation also in those trypanosomes that did not attenuate the ES sufficiently, and which would have resumed growth in the absence of SIF (‘escapers’, Fig 9A). Consequently, when the HD trypanosome population was provided fresh culture medium on either days 1 or 2, the ES-attenuation escapers survived and grew out after 5 days (Fig 10A, washed). This finding was supported by the control experiment using lower starting cell densities, i.e. a 10-fold slowed SIF accumulation (Fig 10B; S13B Fig). In those cultures, SIF would never have accumulated in sufficient amounts to be able to drive the complete population into cell cycle arrest.

An important conclusion that we can draw from the above experiment is that ES-attenuation leads to stumpy formation in less than one day (S7 Fig). Hence, stumpy development in HD cultures in the first two days following induction was exclusively caused by ES-attenuation, and thus independent of SIF.

### SIF and ES-attenuation could feed into the same signaling pathway

**Fig 11:**
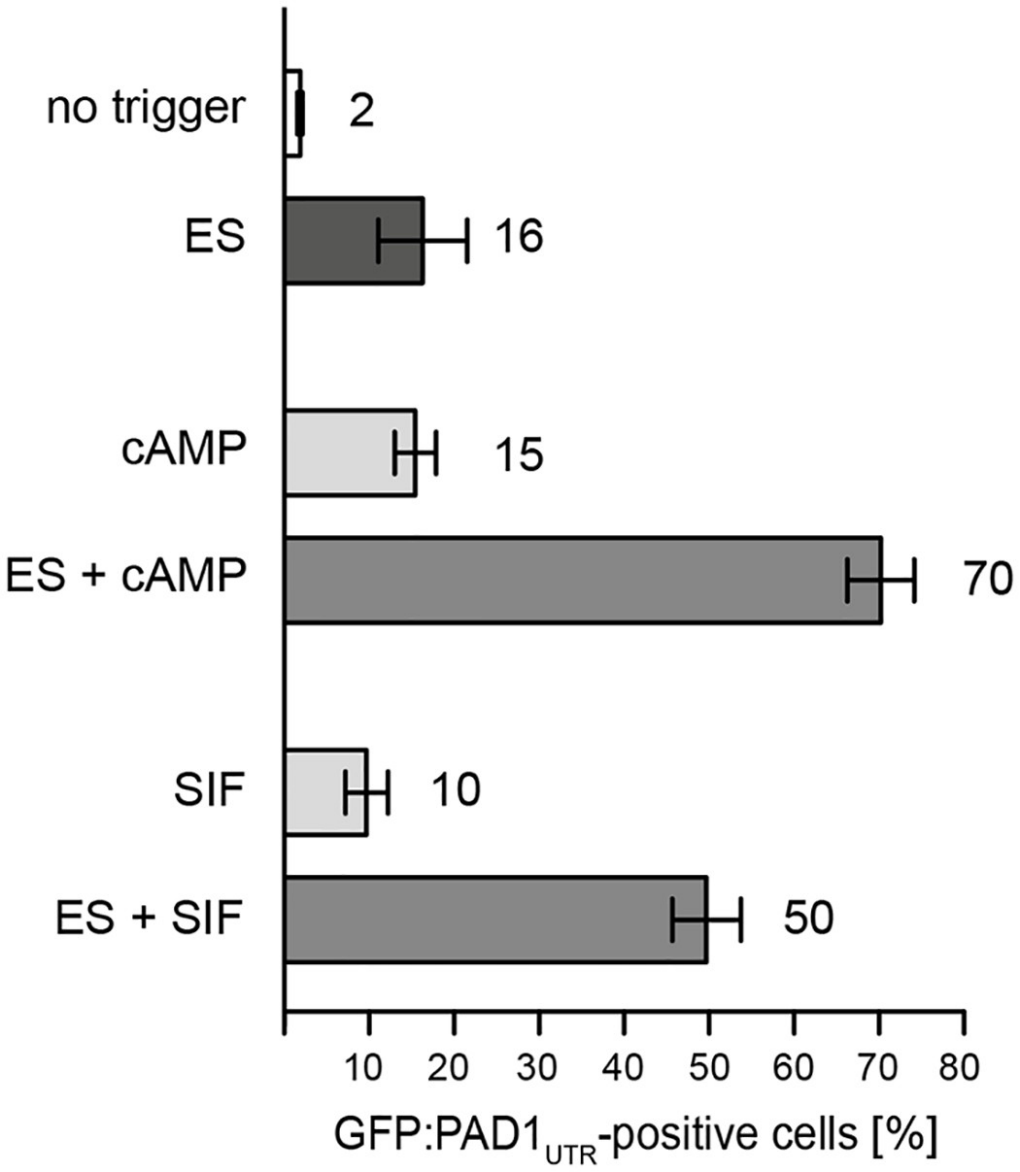
ES attenuation accelerates SIF-induced stumpy formation at the population level. The GFP:PAD1_UTR_ cell line was challenged with ES-attenuation and chemical triggers for stumpy development. The trypanosome culture was adjusted to a cell density of 1x 10^5^ cells/ml and one part was induced with tetracycline (ES). Non-induced cells served as a control (no trigger) and were treated with 200μm cAMP or 0.25x SIF (cAMP, SIF). The last two cultures were tetracycline-induced additionally received the stumpy triggers (ES + cAMP, ES + SIF). After 20 hours, the numbers of GFP:PAD1_UTR_-positive trypanosomes was counted. The values are means ± SD (n > 1200 cells each). ES-attenuation in combination with either SIF or cAMP produces more stumpy cells than the summed effect of the individual cues.

With regard to signal penetration, our experiments suggested that SIF was the dominant differentiation trigger, as ES-attenuation escapers were still responsive to this. We hypothesize that ES-attenuation represents an ‘epigenetic’ signal, downstream of the chemical cue SIF. What, however, happens if the cells receive both triggers, ES-attenuation and SIF, at the same time? To address this question, we exposed cells to both signals, assuming that a population that had been primed with ES-attenuation could react faster to the SIF signal than the non-induced control. We induced VSG overexpression-mediated ES attenuation in the PAD1 reporter cell line, and added SIF or its second messenger cAMP (Fig 11). The combination of ES-attenuation with either of these two chemical signals was by far more effective than each signal alone. Within 20 hours, ES-attenuation and 200 μM cAMP, produced 16% and 15% of GFP:PAD1-positive cells, respectively. When both triggers were combined, 70% of the parasites became stumpy. The combination of SIF and ES-attenuation was also more effective: while SIF alone produced just 10% stumpy parasites, the simultaneous induction of ES attenuation resulted in 50% of trypanosomes being PAD-positive (Fig 11). An extended experiment using different time frames of incubation and different concentrations of the differentiation triggers is shown in S14 Fig.

We tentatively conclude that SIF and ES-attenuation are acting along the same signaling pathway, probably in a cooperative manner. All long slender trypanosomes respond to ectopic VSG overexpression with ES-attenuation. For stumpy development, the ES-activity has to fall below a critical threshold. In the presence of additional SIF this threshold is reached earlier and hence, more trypanosomes can differentiate within the same period. Thus, all our results are compatible with a mechanism, in which VSG-induced ES-attenuation triggers stumpy differentiation in a cell density-independent manner, downstream of the density-dependent quorum sensing factor SIF. Our experiments further underline the multiple roles of the VSG ES as a trypanosome virulence hub. The ES is not only essential for immune evasion and metabolism, but also controls parasite development.

## Discussion

Little is known about the control of *in situ* VSG switching. Basically, there are two possibilities: either the old ES is shut-down and then a new ES is activated, or a new ES is transcriptionally activated before the old one is switched off. Support for the first possibility comes from tagging two ESs with selectable markers [53,54]. In the presence of the drugs rapid switching between the tagged ESs occurred. This suggested that one silent ES lingers in a pre-active state and, thus, is immediately activated once the active ES is silenced. However, another study reported that the inducible block of ES transcription caused growth inhibition and subsequent probing of several silent ESs [13]. This suggested that the silencing of the active ES does not cause an immediate antigenic switch. In addition, depletion of *VSG* mRNA results in a rapid precytokinesis arrest, which suggests that an inactivation of the ES without the simultaneous activation of a new one would be lethal [23]. Therefore, in a previous study, we tested the possibility that a new VSG is activated, before the old one is silenced. This was achieved by inducible overexpression of a second VSG [34]. Surprisingly, the trypanosomes responded with attenuation of the active ES and growth retardation. It is important to note that these cells never stopped growth, i.e. they never arrested in the cell cycle, but rather lingered in a prolonged G1-phase. Interestingly, growth retardation was accompanied by signs of developmental competence. This raised the question whether ectopic VSG overexpression or ES-attenuation could lead to stumpy development. This possibility, however, remained unexplored, as the monomorphic trypanosome strains routinely used in the laboratory are developmentally deficient. Only more natural, pleomorphic parasites are suitable for analyses of trypanosome differentiation [35,36], but large scale cultivation and genetic manipulation are very difficult.

We established the tetracycline-inducible ectopic VSG overexpression system in the pleomorphic strain EATRO 1125 (serodeme AnTat1.1). The induction of ectopic VSG 121 overexpression produced an unexpected phenotypic variability. In a subset of recombinant clones, the ectopic VSG overexpression led to growth arrest. The clones rapidly stopped growing within the first cell division cycle, and did not linger in a prolonged G1-phase, as the monomorphic trypanosomes did. In another subset of clones, however, the pleomorphic trypanosomes did not halt the cell cycle at all, but rather proliferated normally, with only marginally prolonged population doubling times. Initially, we assumed that the latter parasites were simply refractory to VSG induction, but this was not the case. Irrespective of the growth response, all trypanosome clones exchanged the VSG surface coat with similar kinetics, i.e. the endogenous VSG A1.1 was replaced with the ectopic VSG 121. This phenotypic variability was not VSG-dependent. When ectopic VSG 118 overexpression was induced, the VSG coat was exchanged, but only a subset of clones arrested in the cell cycle, while others grew normally. This confirmed that a VSG coat can be readily formed with protein transcribed from outside the active ES. In addition, it showed that the ES-resident VSG can be silenced without shutting off the other parts of the ES. In the proliferating ectopic VSG overexpressors, the VSG promoter-proximal GFP reporter transcripts decrease to about half of wild type levels within 48 hours, and the native VSG A1.1 was silenced over many parasite generations. We propose that such an expression of ESAGs and VSG from two different genomic locations might occur naturally, namely during an *in situ* switch. When the old ES is attenuated, the ESAGs of the new ES will have to functionally complement and, thus, can become limiting for growth. However, the ES-activity can be stalled at levels that still support growth. Consequently, these trypanosomes could potentially survive a switch to a defective or incompatible ES. It has long been known that *T*. *brucei* preferentially populates tissue spaces, enters the brain and, as only recently shown, thrives in fat tissue [55–58]. Assuming that not all ESAGs (or other ES-derived elements) are equally well suited for supporting growth in fat or other tissues, it would be an advantage to probe for the optimal ES and to select for the best adapted parasites as founders of a new population. Additionally, the blood feeding behavior of the tsetse fly is not very choosy and thus, the trypanosomes are confronted with a wide range of hosts. It has long been postulated that host serum compatibility is also a readout of ESAGs, especially ESAGs 6 and 7, which encode the heterodimeric transferrin receptor [59–61]. Thus, any stochastic *in situ* switch could select for the most advantageous ES in a given host [7].

The ectopic overexpression of VSG yielded not only proliferative ectopic VSG overexpressors, but, with similar frequency, VSG overexpressors that rapidly stopped growth once they had attenuated the active ES. We have shown that these clonal populations halt the cell cycle in G1/0. Furthermore, the cells are indistinguishable from the short stumpy life cycle stage in every possible aspect studied. They undergo the same morphological changes, express stumpy marker proteins, including a GFP-reporter for the ‘protein associated with differentiation 1’ (PAD1). They synchronously respond to the differentiation trigger *cis*-aconitate with development to the insect stage, and importantly, they infect the tsetse fly and successfully complete the weeks-long passage through the vector. Thus, the growth arrested VSG overexpressors with an attenuated ES are short stumpy trypanosomes. So far, it has been assumed that the stumpy stage transition is exclusively initiated through the quorum sensing factor SIF, which is continuously secreted by proliferating slender bloodstream trypanosomes [26]. In a paracrine manner SIF is sensed and limits the parasite population size by driving the trypanosomes into G1/0 cell cycle arrest. Thus, the SIF pathway is strictly cell density-triggered [25]. We show that ectopic VSG overexpression induced ES-attenuation yields stumpy stage parasites even at low cell densities and thus, in a SIF-independent way. This happens with very fast kinetics: while SIF-challenged (already committed) slender cells divide between 2 and 3 times before they exit the cell cycle [26,52], ES-attenuation induced stumpy parasites divide just once before they arrest. At this time the ES is attenuated by about 80%. Thus, one or more products from the ES might signal the status of the ES, and initiate the stumpy induction pathway, bypassing the need for high cell density and, thus, SIF. This might be because SIF obviously acts upstream of ES-attenuation, as it is an extracellular cue. By combining SIF and ectopic VSG overexpression induced ES-attenuation we have shown that both triggers work cooperatively. We suggest that a reduction in the ES-activity can prime the parasite for stumpy development, which is triggered once the transcriptional activity drops below the critical threshold. Besides this, we also considered the question whether any cell stress or growth inhibition *per se* could enforce stumpy development. For several reasons the answer is no: first, in monomorphic VSG overexpressors, ES-attenuation precedes growth retardation [34]. Second, cell cycle arrest in pleomorphic parasites, for example due to VSG shortage, does not cause stumpy development [23]. Also, stressing trypanosomes by mild acid treatment does not trigger stumpy differentiation. Lastly, the possibility that stumpy development was caused by nutrient deprivation [62,63], e.g. due to loss of ESAG function, is simply excluded by the fast kinetics of the event: ES-attenuation initiates the developmental progression within one cell cycle, which is clearly faster than expected from a metabolic penalty that depends on the decay of ESAG protein levels.

So why should there be an alternative to cell density-triggered stumpy stage development? In fact, when trypanosomes are injected into natural hosts the parasitemia is very low [56]. It is difficult to envisage how the secreted SIF should accumulate to concentrations that would induce stumpy development, at least outside of tissue spaces. Here, a second, synergistic differentiation trigger could be expedient. All trypanosomes undergoing a transiently or permanently unsuccessful *in situ* switch would contribute to the number of short stumpy cells. Since VSG switching occurs stochastically, ES attenuation-triggered stumpy formation should generate a constant background rate of stumpy differentiation. Such a density independent formation of stumpy parasites has in fact been suggested previously [64]. Based on mathematical simulations the authors state that a density-dependent model cannot explain the observed presence of stumpy cells before SIF reaches an effective concentration. They have termed this the background rate and we surmise that ES-attenuation accounts for this constantly occurring stage transition.

**Fig 12:**
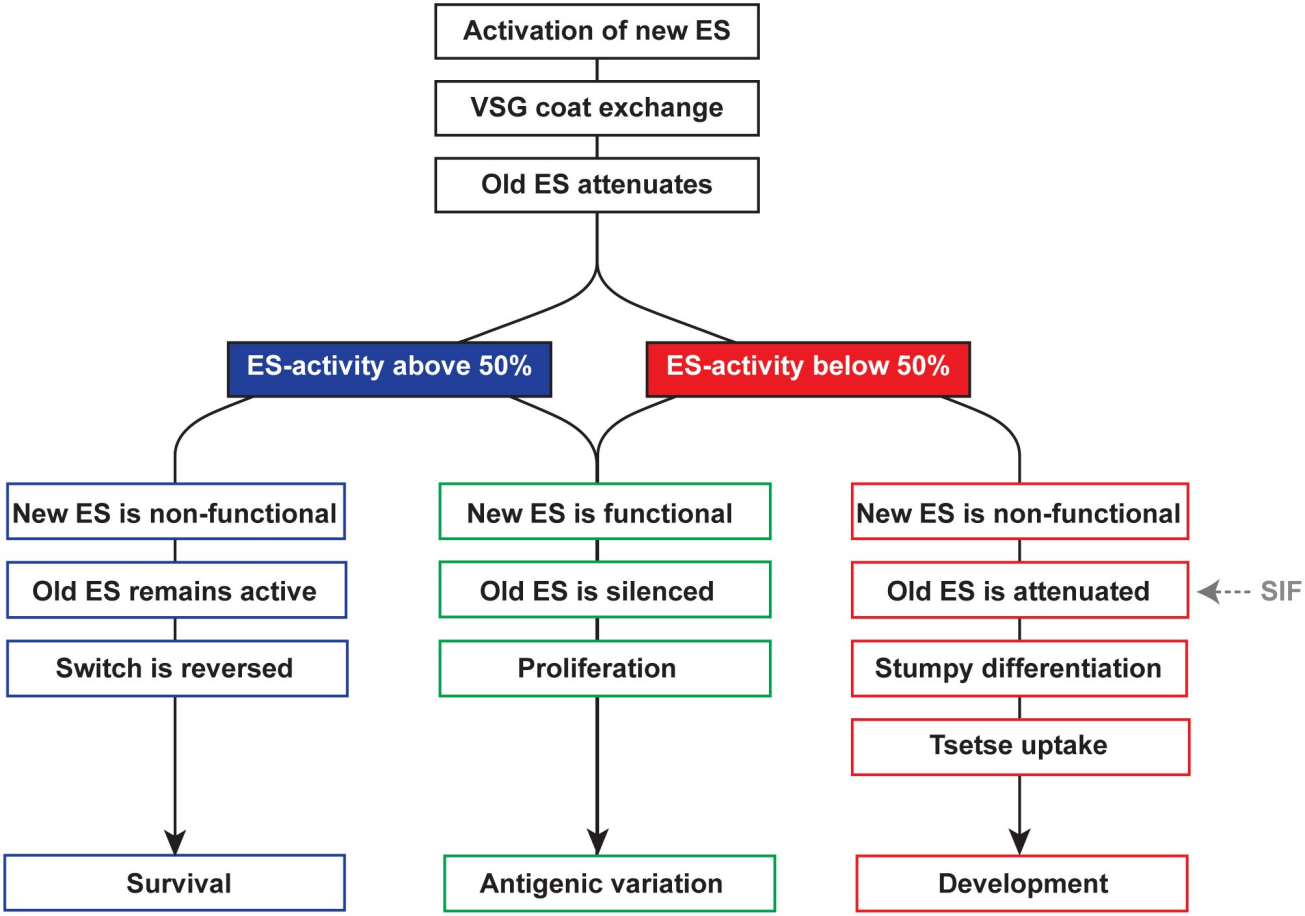
Model of the role of ES-activity for the persistence of *T*. *brucei*. The activation of a new ES during an antigenic *in situ* switch leads to an immediate exchange of the VSG coat, and the attenuation of the old ES is initiated. If the ES-activity remains above 50% (blue) it can functionally complement for a non-functional ES. In this case, the old ES is not silenced further and thus can support growth. If the new ES is functional (green), the old one is silenced and the trypanosomes proliferate. The *in situ* switch is completed. When, however, the ES-activity falls below 50% (red) a non-functional new ES becomes growth-limiting, the trypanosomes arrest growth and differentiate to the tsetse-infective stumpy stage. This developmental transition will be accelerated by the quorum sensing factor SIF. All three scenarios are beneficial for parasite survival on the population level.

All our data are compatible with a model that promotes the VSG ES as a master regulator of antigenic variation and development (Fig 12). When a new ES is activated, the new VSG replaces the old one immediately as the old VSG is transcriptionally silenced within a few hours [34,65]. If the new VSG protein is incompatible or defective, the trypanosome dies, as the parasite cannot form a proper surface coat. When a new VSG coat has been produced, the remainder of the old ES is attenuated, most likely by epigenetic mechanisms [13,34]. When the old ES is attenuated to a critical threshold the ES-transcripts become limiting. This shortage has to be compensated by the ESAGs of the newly activated ES. If the complementation is successful, the old ES is silenced, and the antigenic switch is completed. If, however, elements of the new ES are defective or incompatible, the trypanosomes can react in two ways. If the ES transcript levels remain above 50%, the cells stop further attenuation of the old ES, which allows the parasites to keep proliferating. If, however, the ES is silenced to levels below 50%, the irreversible transition to the stumpy life cycle stage is initiated and the trypanosomes arrest the cell cycle, thereby becoming fully competent for tsetse fly transmission. Thus, the parasites do not necessarily die when an ES is activated that does not provide a good complement of ESAGs (or other essential ES transcripts, such as small RNAs). Instead, a ‘rescue program’ is launched ensuring the survival of the trypanosome population. Although it is not straightforward to experimentally test this hypothesis, our data make it even more difficult to exclude it.

## Materials and methods

### Trypanosome cultivation

All generated cell lines were based on the pleomorphic *Trypanosoma brucei brucei* strain EATRO 1125 (AnTat1.1 13–90) expressing VSG AnTat1.1, abbreviated as VSG A1.1 [35,66]. The bloodstream form parasites were cultured in HMI-9 medium, supplemented with 10% (v/v) fetal bovine serum and 1.1% (w/v) methylcellulose to increase viscosity (Sigma-94378), at 37°C and 5% CO_2_ [67,68]. To maintain expression of the T7-polymerase and the tetracycline repressor, cells were cultivated in the presence of 2.5 μg/ml hygromycin and 1.25 μg/ml G418. Trypanosome cultures were strictly kept at densities below 5x 10^5^ cells/ml in order to prevent developmental transition to the stumpy stage. To harvest the cells, methylcellulose had to be removed from the cultures. Therefore, we first diluted the cultures at least 1:4 with sterile trypanosome dilution buffer (TDB; 5 mM KCl, 80 mM NaCl, 1 mM MgSO_4_, 20 mM Na_2_HPO_4_, 2 mM NaH_2_PO_4_, 20 mM glucose, pH 7.6). Next, the diluted culture was filtered (MN 615 1/4, Macherey-Nagel, Germany) using sterile conditions and centrifuged (1 500 x*g*, 15 minutes, RT). The AMAXA Nucleofector II (Lonza, Switzerland) was used to transfect 3x 10^7^ trypanosomes with 10 μg of linearized plasmid DNA. Transgenic clones were selected by serial dilution and the addition of the respective antibiotics.

Monomorphic parasites of the *Trypanosoma brucei brucei* strain Lister 427 (MITat1.6 wild type cells expressing VSG 121 or MITat1.2 wild type cells expressing VSG 221) were cultivated at 37°C and 5% CO_2_ in HMI-9 containing 10% (v/v) fetal bovine serum.

### Generation of cell lines

For tagging of the active VSG expression site the open reading frame of an eGFP was inserted upstream of the puromycin resistance cassette into the pLF12 plasmid [12]. The resulting construct was targeted to the ES promotor region and consisted of the *eGFP* flanked by aldolase UTRs and the puromycin resistance cassette with actin 5´ and aldolase 3´UTRs. The plasmid was linearized with KpnI and SacI and transfected into the parental AnTat1.1 13–90 cell line (A1.1^ES^). Selection with 1 μg/ml puromycin yielded the GFP^ESpro^A1.1^ES^ cell line. To generate the GFP:PAD1_UTR_ stumpy reporter cell line, the plasmid p4231 (courtesy of M. Carrington; [34]) was transfected into AnTat1.1 13–90 cells (A1.1^ES^). This construct consists of a *GFP* sequence with a nuclear localization signal that is followed by the 3´UTR of *PAD1*. As the stumpy stage specific transcript increase of *PAD1* is controlled by parts of its 3´UTR [37], early stumpy development can be monitored by the appearance of green fluorescent nuclei in the GFP:PAD1_UTR_A1.1^ES^ cell line.

For ectopic overexpression of VSG 121, the reporter cell lines were transfected with the NotI-linearised pRS.121 plasmid [34], giving rise to the cell lines GFP^ESpro^A1.1^ES^121^tet^ and GFP:PAD1_UTR_A1.1^ES^121^tet^. For ectopic overexpression of VSG 118 (kindly provided by N. Jones), the *VSG 118* open reading frame, flanked by its wild type 3´UTR and the EP 5´UTR, was inserted into the pLew82v4 vector (24 009; Addgene plasmid). The construct was linearized with NotI and transfected into the GFP:PAD1_UTR_ cell line, giving rise to the GFP:PAD1_UTR_A1.1^ES^118^tet^ cell line.

### Serial dilution of outgrowing ectopic VSG overexpressors

Parasites were diluted to a concentration of 25, 250, 2,500 or 25,000 cells/ml and ectopic VSG overexpression was induced by the addition of tetracycline (1 μg/ml). Subsequently, the dilution was transferred to a 96-well microtiter plate, each well containing 200 μl. Thus, every well of the plate contained 5, 50, 500 or 5,000 cells. As a control, non-induced long slender cells were seeded at a concentration of 5 cells per well. As the medium color shifts from pink (alkaline) to orange (acidic) at high cell densities the outgrown wells were readily identified by changes of medium color after 18 days of incubation. To estimate the number of outgrowing cells we assumed that a single cell is able to establish an outgrowing population. Thus, the number of outgrowing cells was calculated by dividing the amount of seeded ectopic VSG overexpressors per plate by the number of outgrown wells.

### Mild acid treatment of slender parasites

Slender parasites of the GFP:PAD1_UTR_A1.1^ES^ cell line were harvested as described above and transferred to liquid HMI-9 (pH 7 or pH 5.5). The cells were incubated for 30 minutes or two hours in the medium at 37°C. Subsequently, 1 μl propidium iodide (1 mg/ml) was added to 1 ml culture and analyzed with a BD Bioscience FACSCalibur Flow Cytometer. 20,000 cells were counted per sample and the data were analyzed with the BD CellQuest Pro Software (BD Bioscience, USA). An aliquot of the pH treated cells was washed two times with TDB and further cultivated in HMI-9, supplemented with methylcellulose. Population growth was recorded for 48 hours after treatment, and the expression of the GFP:PAD1_UTR_ was monitored.

### SIF-induced differentiation to the stumpy stage

For the generation of density-induced stumpy parasites, slender cells at a seeding density of 5x 10^5^ cells/ml were cultivated without dilution for 48 hours. This allowed the accumulation of SIF and subsequent stumpy formation.

To analyze the impact of SIF on growth arrested ectopic VSG overexpressors, the parasites were treated either with a SIF concentrate or the downstream analogue pCPT-cAMP (Sigma-Aldrich, USA). To generate the SIF concentrate, monomorphic parasites at a density of 5x 10^4^ cells/ml were grown for 50–52 hours to maximum cell density (0.7-1x 10^7^ cells/ml). Subsequently, filtration was used to remove the cells and proteins were depleted from the supernatant via methanol precipitation. The protein free medium was lyophilized and resuspended to an x-fold concentration, whereby 1x corresponds to conditioned medium without further concentration steps.

The SIF concentrate was diluted in pre-warmed TDB to a concentration of 1x or 1.5x and the SIF downstream analogue pCPT-cAMP to 400 or 800 μM. Parasites were diluted to a concentration of 1x 10^5^ cells/ml, ectopic VSG overexpression and, thus, ES-attenuation was induced by the addition of tetracycline (1 μg/ml). Immediately, 1.5 ml of the recently induced trypanosomes were transferred to a 24-well plate. To each well 500 μl of the dissolved compounds or TDB alone was added (control). Thus, SIF had a final concentration of 0.25x or 0.37x and pCPT-cAMP of 100 μM or 200 μM. In parallel, non-induced slender cells of the same strain were treated identically to the ectopic VSG overexpressors. Subsequently the parasites were incubated at 37°C and 5% CO_2_. After 20 and 28 hours of incubation the amount of GFP:PAD1_UTR_-positive cells was microscopically analyzed.

### Differentiation to the procyclic stage

Trypanosomes ectopically overexpressing VSG 121 for 48 hours (ES-attenuation-induced stumpy parasites), non-induced slender or density-induced stumpy cells were harvested from HMI-9 supplemented with 1.1% (w/v) methylcellulose. To trigger differentiation to the procyclic stage the parasites were resuspended in DTM culture medium [47] to a density of 2x 10^6^ cells/ml. After the addition of 3 mM *cis*-aconitate and 3 mM citrate, the cultures were incubated at 27°C (CCA treatment).

Samples were collected after 0, 6 or 24 hours of CCA treatment. The detection of EP1 was conducted as described by Batram et al., 2014 using an Alexa647-conjugated anti-mouse antibody for FACS analyses and an Alexa594 conjugated anti-mouse antibody for the acquisition of microscopic images [34]. A BD Bioscience FACSCalibur Flow Cytometer was used for flow cytometry and 20,000 cells were counted for every sample. The data were analyzed with the BD CellQuest Pro Software (BD Biosience, USA).

### Maintenance, infection and dissection of tsetse flies

Tsetse flies *(Glossina morsitans morsitans)* were kept at 27°C and a relative humidity of 70%. The insects were fed twice a week through a silicon membrane with defibrinated sterile sheep blood (ACILA, Germany). After a maximum of 48 hours post-eclosion, the flies were infected with trypanosomes during their first blood meal. For this, the parasites were harvested and resuspended to a density of 2x 10^6^ cells/ml in blood supplemented with 60 mM *N*-acetylglucosamine. 50 flies each were fed with density-induced stumpy trypanosomes of the parental GFP:PAD1_UTR_A1.1^ES^ cell line or with parasites ectopically overexpressing VSG 121 for 56 hours (ES-attenuation-induced stumpy parasites). After >50 days of infection the surviving flies were starved for at least 24 hours, before they were dissected as described by Rotureau et al., 2011 [69]. First, the salivary glands were isolated immediately after dissection. Then, the complete tsetse alimentary tract was dissected in a drop of PBS and microscopically examined for the presence of trypanosomes (density-induced: 44 flies; ES-attenuation-induced: 47 flies). Next, tsetse foregut and proventriculus were separated from the midgut in different drops of PBS and parasites were released from the tissues. Subsequently, immunostaining was performed as described below.

### RNA and protein analyses

Total RNA was extracted from 1x 10^8^ trypanosomes using the RNeasy Mini Kit (Qiagen, Netherlands). For fluorescent labeling, 3 μg of glyoxal-denaturated RNA was transferred to a nitrocellulose membrane using a Minifold Dotblotter (Schleicher & Schuell, Germany). The blots were hybridized over night at 42°C with oligonucleotide probes coupled to IRDye 682 (VSG 121: GCTGCGGTTACGTAGGTGTCGATGTCGAGATTAAG; VSG AnTat1.1: GTCTTTCTCTTCTTTCCCTTTGCACTTTTC) or IRDye 782 (tubulin: TCAAAGTACACATTGATGCGCTCCAGCTGCAGGTC). For radioactive quantifications the denatured RNA was separated on an agarose gel and transferred to a nylon membrane. GFP mRNA was detected with a ^32^P-labeled probe (complete eGFP ORF, Thermo Scientific DecaLabel DNA Labeling Kit) and quantified using a Phosphorimager.

Protein samples were prepared by acetone precipitation and analyzed via protein dot-blotting (6x 10^5^ cell equivalents) as described by Batram et al., 2014 [34], or on Western blots (2x 10^6^ cell equivalents). After blocking with 5% (w/v) milk powder in PBS, the primary antibodies were diluted in PBS containing 1% (w/v) milk powder and 0.1% (v/v) Tween 20: rat anti-VSG AnTat1.1 (VSG A1.1) 1: 20,000 [35]; rabbit anti-VSG 121 1:2,000 (courtesy of M. Carrington); rabbit anti-LipDH 1: 10,000 [70]; rabbit anti-PIP39 1:750 (courtesy of B. Szoor), rabbit anti-H3 1:10,000 [71]; guinea pig anti-H3 1:5,000 [72]; mouse anti-PFR (L13D6) 1:200 [73]. Species-specific, IRDye coupled secondary antibodies (LI-COR Biosciences, Netherlands) were used for infrared detection of proteins (1:10,000 in PBS containing 1% (w/v) milk powder and 0.1% (v/v) Tween 20). Analyses and quantification of fluorescently labeled protein and RNA was conducted using the Licor Odyssey Infrared Imaging System (LI-COR Biosciences, Netherlands).

### mRNA FISH

To quantify mRNA levels via FISH the QuantiGene ViewRNA ISH Cell Assay kit (Affymetrix, USA) was used, essentially following the manufacturer’s instructions. At least 1x 10^7^ trypanosomes were harvested, fixed with 4% (w/v) formaldehyde (FA) for 10 minutes at room temperature and, subsequently, washed two times with PBS. Cells were allowed to settle on poly-l-lysine-coated slides (within hydrophobic circles) for 30 minutes. For protease digestion the settled cells were incubated with the protease solution (1:1 600 in PBS) for 15 minutes at 25°C. The following probes for mRNA detection were used in a 1:100 dilution of the original stock: *eGFP* (full antisense ORF, red = type 1) and *ESAG6* (antisense to nucleotides 107–1206 Tb427.BES40.3, red = type 1). Only samples from the same slide were compared for quantification of mRNA levels. Per slide, fixed cells (non-induced slender, density-induced stumpy and ectopic VSG overexpression induced for 24 and 48 hours) of a proliferating or growth arrested clone were incubated either with the *ESAG6* or *eGFP* probe.

### Fluorescence staining

The mitochondria of the parasites were visualized by incubation of trypanosome cultures with 50 nM MitoTracker Red CMXRos (ThermoFisher Scientific, USA) for 20 minutes at 37°C. The cells were then harvested as described above, washed with TDB and chemically fixed for 15 minutes at room temperature with 2% (w/v) formaldehyde (FA) and 0.05% (v/v) glutaraldehyde in PBS.

For the detection of VSGs chemically fixed parasites (30 minutes, 2% (w/v) FA) were allowed to settle on poly-l-lysine-coated slides. The cells were blocked for 30 minutes with 1% (w/v) BSA and incubated with a rat anti-VSG AnTat1.1 (1:4,000, [35]) and a rabbit anti-VSG 121 or anti-VSG 118 (1:500) antibody diluted in 0.1% (w/v) BSA in PBS. Alexa488- and Alexa594-conjugated anti-rabbit and anti-rat antibodies, were used at dilutions of 1:500 (in PBS containing 0.1% (w/v) BSA; ThermoFisher Scientific, USA). For flow cytometric examination of ectopic VSG 121 expression, cells were blocked and stained with the rabbit anti-VSG 121 (1:500) in suspension. An Alexa647-conjugated anti-rabbit antibody was used (1:500 in PBS containing 0.1% (w/v) BSA; ThermoFisher Scientific, USA) and 20,000 cells per sample were counted with a BD Bioscience FACSCalibur Flow Cytometer. The data were analysed with the BD CellQuest Pro Software (BD Bioscience, USA). For the detection of PAD1, fixed cells were permeabilized for 20 minutes with 0.05% (v/v) Triton X-100 and incubated with PBS containing 20% (v/v) FCS for 45 minutes. Next, a rabbit anti-PAD1 antibody (1:100 in PBS containing 20% (v/v) FCS, [32]) was added, followed by incubation with an Alexa594-conjugated anti-rabbit antibody (1:500 in PBS containing 20% (v/v) FCS; ThermoFisher Scientific, USA).

Parasites isolated from tsetse flies were spread on poly-l-lysine-coated slides, dried and fixed for 30 seconds in ice-cold methanol. After rehydration for 15 minutes in PBS a mouse monoclonal anti-PFR antibody (L8C4, 1:20 in PBS containing 0.1% (w/v) BSA, [73]) was added to the cells and an Alexa594-conjugated anti-mouse antibody (1:500 in PBS containing 0.1% (w/v) BSA; ThermoFisher Scientific, USA) was used as secondary antibody.

### Microscopy

Images were acquired using the iMIC wide field fluorescence microscope (FEI—TILL Photonics, Germany), equipped with a CCD camera (Sensicam qe, pixel size 6.45 μm, PCO, Germany). Z-stack images were recorded using 100x (NA 1.4) or 60x (NA 1.45) objectives (Olympus, Germany) and the filter cubes ET-mCherry-Texas-Red, ET-GFP and DAPI (Chroma Technology CORP, USA). All equipment was controlled with the ‘Live acquisition’ software (TILL Photonics, Germany). The 3D images consisting of 100 slices with a z-step size of 100 nm are displayed as maximum intensity projections (ImageJ). For signal quantifications images were deconvolved using Huygens Essential software (Scientific Volume Imaging B. V., Netherlands) and intensities were measured in Z-projections (method sum slices). Alternatively, cells were recorded at 100x magnification using the DMI6000B wide field fluorescence microscope (LEICA microsystems, Germany), equipped with a DFC365FX camera (pixel size 6.45 μm, LEICA microsystems, Germany). DIC images are average projections of 10–20 slices with a z-step size of 67 nm and fluorescent images maximum intensity projection. Images are shown in false colors with green fluorescence in green, blue in grey and red in magenta to ensure the availability of color information for individuals with color vision deficiencies [74]. Pseudocoloring, intensity projections and intensity measurements were performed using ImageJ software (National Institutes of Health).

## Supporting information

S1 Fig Schematic illustration of the AnTat1.1 ES with an inserted GFP open reading frame downstream of the ES-promotor (GFP^ESpro^A1.1^ES^). As the order of ESAGs (1–8) in the AnTat1.1 ES is unknown the consensus succession is shown [14]. PURO, puromycin resistance; arrow, ES-promoter.
(TIF)

S2 Fig Cumulative growth curves of the ectopic VSG overexpressors of Fig 1. Representative cumulative growth curves of (A) the GFP_ESpro_A1.1_ES_121_tet_ and (B) the GFP:PAD1_UTR_A1.1_ES_121_tet_ cell lines are shown. Tetracycline-induced (triangles) and non-induced (squares) cells of proliferating (left) and growth arrested clones (right) were analysed. Data are means (± SD) of three experiments. Due to the small standard deviation, the error bars are not visible. The parental AnTat1.1 cell line (circles) served as a growth control.
(TIF)

S3 Fig Flow cytometry analysis of ectopic VSG 121 expression. A proliferating clone of the GFP:PAD1_UTR_A1.1^ES^121^tet^ cell line was used for immunostaining and subsequent FACS analysis. Non-induced cells (0 h) and VSG overexpressing parasites induced for 24 hours were stained with an antibody against the ectopic VSG 121. The parental AnTat1.1 wild type cell line served as a negative control.
(TIF)

S4 Fig Ectopic overexpression of VSG 118 causes distinct growth phenotypes. Representative growth curves of tetracycline-induced (triangles) and non-induced (squares) cells of (A) proliferating and (B) growth arrested clones. The parental AnTat1.1 cell line (circles) served as a growth control. Data are means (± SD) of three experiments. Due to the small standard deviation, the error bars are not visible. (C) Immunofluorescence analysis of a proliferating clone using antibodies against the ectopic VSG 118 (magenta) and the endogenous VSG A1.1 (green). Non-induced cells (upper panel) as well as cells induced for 24 hours (lower panel) were analyzed. DNA was stained with DAPI (grey). Scale bar: 20 μm.
(TIF)

S5 Fig The transcriptional status of the active ES is different in arrested and proliferating ectopic VSG 121 overexpressors. Northern blot analyses of total RNA samples of (A) a growth arrested and (B) a proliferating clone of the GFP^ESpro^ reporter cell line. *GFP* transcripts of cells ectopically overexpressing VSG 121 for up to 48 hours were detected with a ^32^P-labeled probe and the signals were quantified with a Phosphorimager. Fluorescently labeled 18s rRNA was used for normalization. The signal ratio *GFP*/*rRNA* was set to 1 for the non-induced samples. The parental AnTat1.1 13–90 cell line served as a control.
(TIF)

S6 Fig The ectopic VSG 121 is expressed for extended periods in proliferating ectopic VSG overexpressors. Immunofluorescence analysis of a proliferating clone of the GFP:PAD1_UTR_ reporter cell line using antibodies against the ectopic VSG 121 (magenta, left) and the endogenous VSG A1.1 (green, middle). Non-induced cells (0 days) as well as cells induced for 7 and 28 days were analyzed. The merged antibody signal is shown on the right panel. DNA stained with DAPI (grey) is represented in the merged image only. Scale bar: 20 μm.
(TIF)

S7 Fig Stumpy reporter expression, mitochondrial branching and PIP39 expression in a growth arrested ectopic VSG overexpressor. A growth arrested clone of the GFP:PAD1_UTR_ reporter cell line was analyzed. Non-induced slender (0 h) or density-induced stumpy cells (st) of the same clone served as controls. (A) Trypanosomes were microscopically analyzed for the presence of the green fluorescent GFP:PAD1_UTR_ reporter after 24 and 48 hours of ectopic VSG overexpression. Values are given as percentages (± SD) of two experiments (total n > 500). (B) Quantification of 1K1N cells possessing a branched mitochondrion after 24 and 48 hours of ectopic VSG overexpression. The mitochondrion was stained with mitotracker prior to fixation and DAPI staining. Values are given as percentages (± SD) of three experiments (total n > 600). (C) Western blot stained with an antibody against a glycosomal DxDxT class phosphatase (PIP39, green), whose expression increases during density-induced stumpy development (st). PIP39 is upregulated within 48 hours of ectopic VSG overexpression. Detection of paraflagellar rod (PFR) proteins served as a loading control (magenta).
(TIF)

S8 Fig pH-stress does not cause stumpy development. Slender parasites of the GFP:PAD1_UTR_ reporter cell line were incubated in HMI-9 medium at pH 7 or pH 5.5 for (A) 30 minutes or (B) 2 hours. To determine cell viability, the parasites were stained with propidium iodide and analyzed via flow cytometry. Within 30 minutes of incubation at pH 5.5 the majority of the cells had died. After 2 hours no living parasites were detectable. To determine if the cells, which were still viable after 30 minutes of pH-stress, had arrested in the cell cycle and differentiated to the stumpy stage, the culture was washed two-times with TDB and further incubated in HMI-9 at pH 7, supplemented with methylcellulose. (C) Parasite growth was monitored for 48 hours after 30 minutes of treatment at pH 5.5. The mild acid treated cells grew with the same doubling times as the pH 7 control, and hence, had not differentiated. Data are means (± SD) of experiments performed in triplicate. Due to the small standard deviation, the error bars are not visible. (D) The number of GFP:PAD1_UTR_-positive cells was determined microscopically after 24 and 48 hours of pH treatment for 30 minutes. Values are presented as percentages of cells (± SD) of triplicate experiments (total n > 600 cells). Due to the small standard deviation the error bars are not visible.
(TIF)

S9 Fig Neither stumpy reporter expression nor mitochondrial branching can be detected in a proliferating ectopic VSG overexpressor. A proliferating clone of the GFP:PAD1_UTR_ reporter cell line was analyzed after 24 and 48 hours of ectopic VSG overexpression. Non-induced slender (0 h) or density-induced stumpy cells (st) of the same clone served as controls. (A) Trypanosomes were microscopically analyzed for the presence of the green fluorescent GFP:PAD1_UTR_ reporter. Values are given as percentages (± SD) of two experiments (total n > 700). (B) Quantification of 1K1N cells possessing a branched mitochondrion. The mitochondrion was stained with mitotracker prior to fixation and DAPI staining. Values are given as percentages (n > 250).
(TIF)

S10 Fig SIF-induced stumpy development of the parental AnTat1.1 cell line. Slender AnTat1.1 parasites were cultivated without dilution. Two different starting cell densities, 5x 10^4^ cells/ml (light grey circles) and 2.5x 10^5^ cells/ml (dark grey circles) were used. Data are means (± SD) of three experiments.
(TIF)

S11 Fig Non-cumulative growth curves of re-induced ectopic VSG overexpressors and of parasites induced for the first time (Fig 9). Analyses of a growth arrested clone of the GFP:PAD1_UTR_ reporter cell line that resumed growth. (A) Representative growth curve of parasites induced for the first time (triangles) and non-induced (squares) cells. Data are means (± SD) of two experiments. (B) After 48 hours of ectopic VSG overexpression tetracycline was removed. Then, the parasites were further cultivated without tetracycline for one week. The growth was record once tetracycline was re-added to the culture (grey triangles). Parasites of the same clone, which were cultivated for the same time and had never been induced with tetracycline, were induced for the first time (white triangle) as a control. Data are means (± SD) of three experiments.
(TIF)

S12 Fig The cell surface coat of outgrowing ectopic VSG 121 overexpressors is still dominated by the ectopic VSG 121. Immunofluorescence analysis of a growth arrested clone of the GFP:PAD1_UTR_ reporter cell line using antibodies against the ectopic VSG 121 (magenta, left) and the endogenous VSG A1.1 (green, middle). Non-induced cells (0 days) as well as cells induced for 7 and 28 days were analyzed. The merged antibody signal is shown on the right panel. DNA stained with DAPI (grey) is displayed in the merged image only. Scale bar: 20 μm.
(TIF)

S13 Fig Non-cumulative growth curves of washed ectopic VSG overexpressors (Fig 10). Growth curves were recorded to analyse the impact of SIF on a growth arrested ectopic VSG overexpressor. To determine at which time point the secreted SIF affected the ectopic VSG overexpressors, SIF was removed from the cultures after 2 days of induction by washing (washed at day 2 +tet). Induction was maintained due to the re-addition of tetracycline and cultures were diluted again once they resumed growth. Data are means (± SD) of three experiments. (A) High parasite density (HD, 2.5x 10^5^ cells/ml) allowed the accumulation of SIF during ectopic VSG overexpression. (B) SIF induced stumpy development was prevented at low parasite density (LD, 2.5x 10^4^ cells/ml). (C, D) Non-induced cells (C, -tet) and parasites of the parental AnTat1.1 cell line (D, control) were treated in the same way to verify that washing of the cells did not affect growth.
(TIF)

S14 Fig The combined effect of ES-attenuation and classical triggers for stumpy development. Either non-induced cells (-tet) or cells induced for ES-attenuation (addition of tetracycline, +tet) were challenged at a starting cell density of 1x 10^5^ cells/ml with SIF (A, B) or pCPT-cAMP (C, D). SIF and pCPT-cAMP were both used in two different concentrations that provided a good window for observation. Non-induced and induced cells were analyzed without adding the stumpy differentiation triggers as controls. The amount of GFP:PAD1_UTR_-positive cells was determined microscopically after 20 and 28 hours of treatment. Values are presented as percentages of cells (± SD) of a triplicate experiment (total n > 600 cells). The data after 20 hours of treatment with 200 μM pCPT-cAMP or 0.25xSIF are merged in Fig 11 with a second experiment. After 20 hours of exposure to the lower SIF concentration almost 6-fold more parasites (0.25x SIF +tet: 46%) expressed the stumpy reporter than in the non-induced cells (0.25x SIF -tet: 8%). With the higher SIF concentration 2-fold more were GFP:PAD1_UTR_-positive than in the non-induced cells (0.37x SIF +tet: 59%; 0.37x SIF -tet: 22%). In the absence of SIF (control) 2% of the non-induced and 20% of the ectopic VSG overexpressors became stumpy. After 28 hours of incubation with 0.25x SIF, about twice as many cells (91%) expressed the stumpy reporter than in the non-induced parasites (0.25x SIF -tet: 42%). Without additional SIF (control +tet) ES-attenuation alone yielded 50% stumpy cells. Thus, the combination of SIF and ES-attenuation generated a pure stumpy population faster than each trigger alone. This was confirmed with the downstream signal cAMP, with the presence of the additional stumpy-differentiation triggers also causing an increase in the number of stumpy ectopic VSG 121 overexpressors.
(TIF)
