# Supplementary figures and images for: A quorum sensing-independent path to stumpy development in Trypanosoma brucei
Source: PLoS Pathog. 2017 Apr 10;13(4):e1006324. doi: 10.1371/journal.ppat.1006324 (PMC5398725; doi:10.1371/journal.ppat.1006324)

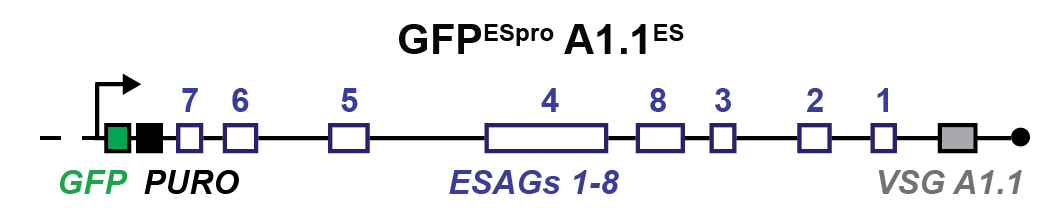

Supplement: S1 Fig — As the order of ESAGs (1–8) in the AnTat1.1 ES is unknown the consensus succession is shown [14]. PURO, puromycin resistance; arrow, ES-promoter. (TIF) [file ppat.1006324.s001.tif]

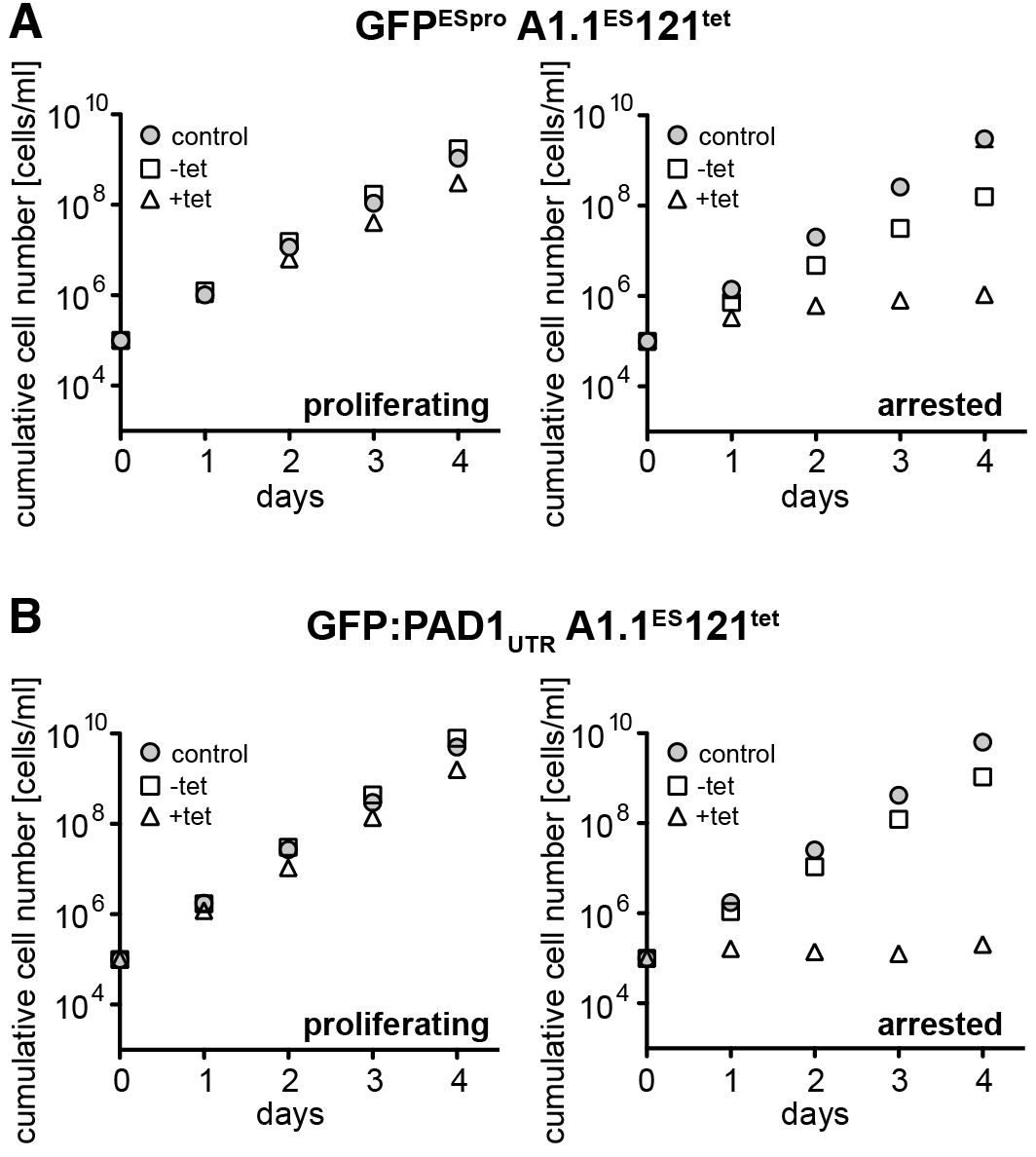

Supplement: S2 Fig — Representative cumulative growth curves of (A) the GFPESproA1.1ES121tet and (B) the GFP:PAD1UTRA1.1ES121tet cell lines are shown. Tetracycline-induced (triangles) and non-induced (squares) cells of proliferating (left) and growth arrested clones (right) were analysed. Data are means (± SD) of three experiments. Due to the small standard deviation, the error bars are not visible. The parental AnTat1.1 cell line (circles) served as a growth control. (TIF) [file ppat.1006324.s002.tif]

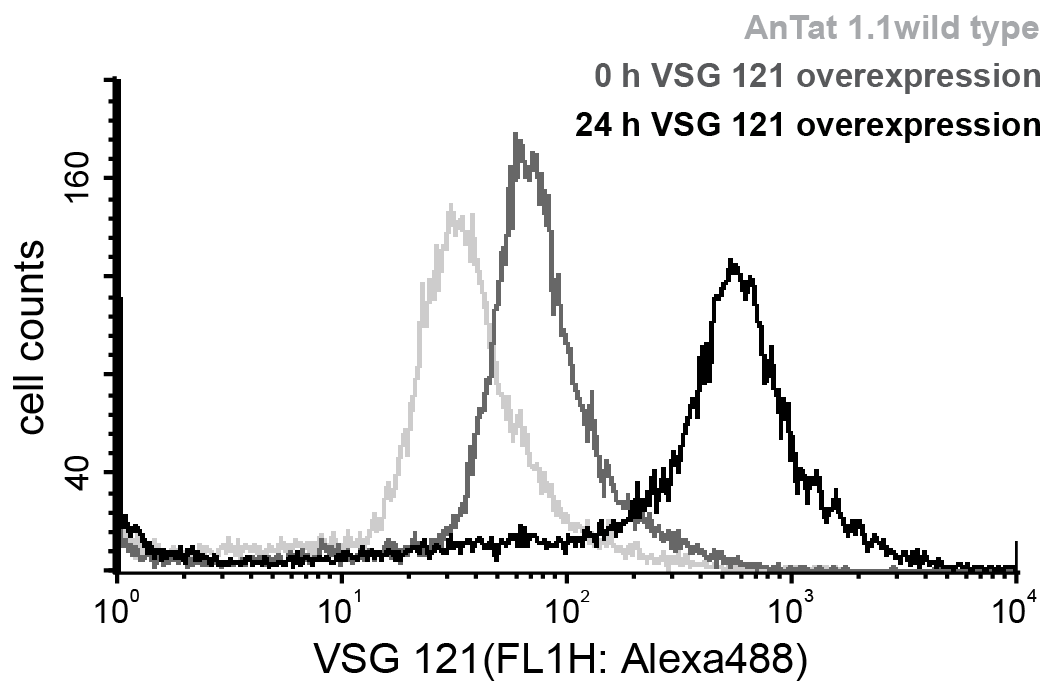

Supplement: S3 Fig — A proliferating clone of the GFP:PAD1UTRA1.1ES121tet cell line was used for immunostaining and subsequent FACS analysis. Non-induced cells (0 h) and VSG overexpressing parasites induced for 24 hours were stained with an antibody against the ectopic VSG 121. The parental AnTat1.1 wild type cell line served as a negative control. (TIF) [file ppat.1006324.s003.tif]

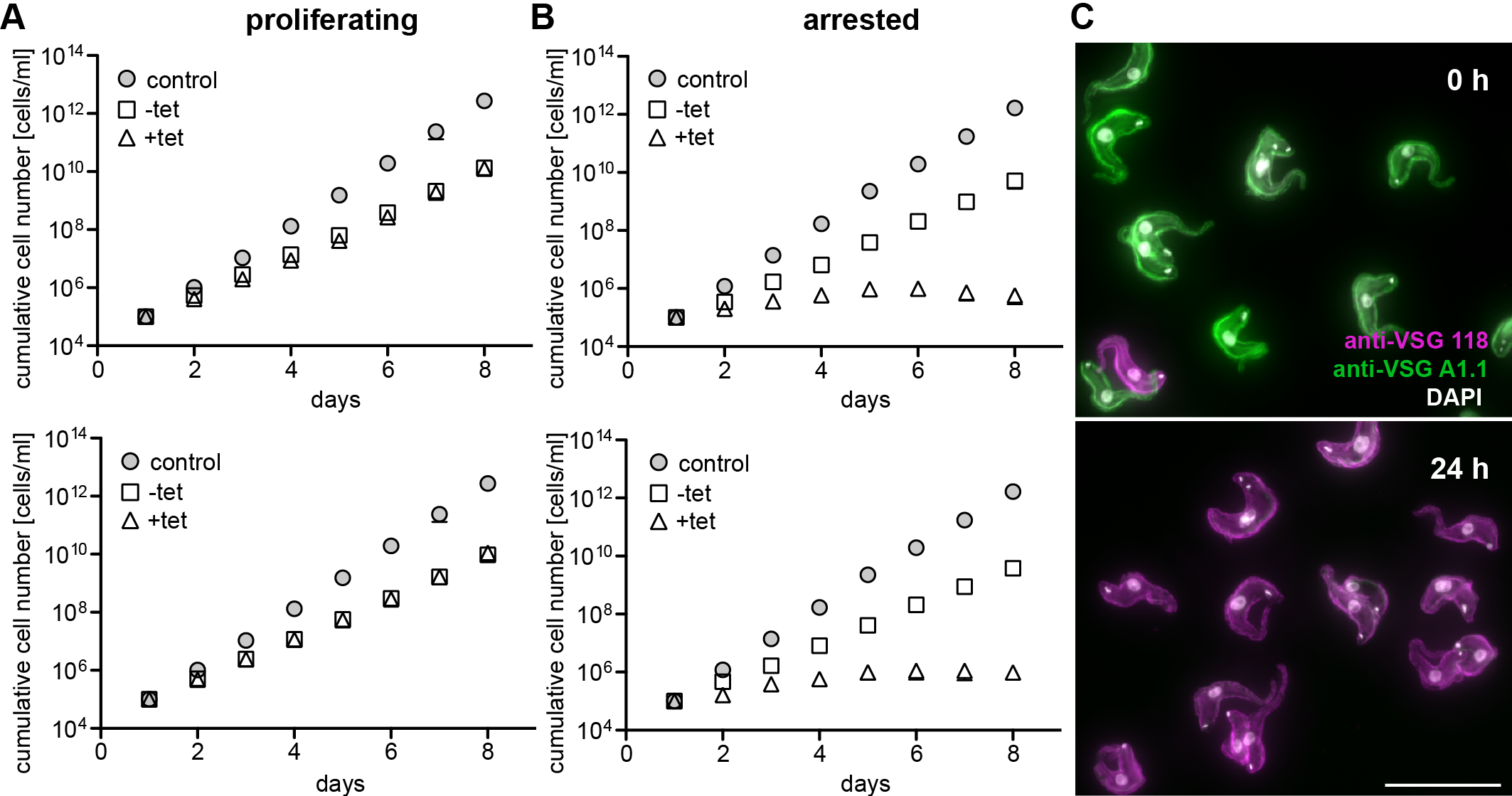

Supplement: S4 Fig — Representative growth curves of tetracycline-induced (triangles) and non-induced (squares) cells of (A) proliferating and (B) growth arrested clones. The parental AnTat1.1 cell line (circles) served as a growth control. Data are means (± SD) of three experiments. Due to the small standard deviation, the error bars are not visible. (C) Immunofluorescence analysis of a proliferating clone using antibodies against the ectopic VSG 118 (magenta) and the endogenous VSG A1.1 (green). Non-induced cells (upper panel) as well as cells induced for 24 hours (lower panel) were analyzed. DNA was stained with DAPI (grey). Scale bar: 20 μm. (TIF) [file ppat.1006324.s004.tif]

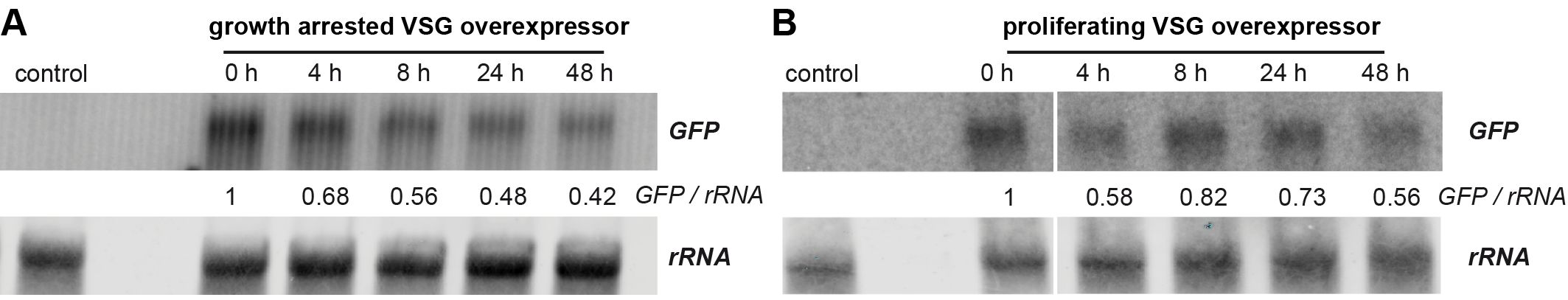

Supplement: S5 Fig — Northern blot analyses of total RNA samples of (A) a growth arrested and (B) a proliferating clone of the GFPESpro reporter cell line. GFP transcripts of cells ectopically overexpressing VSG 121 for up to 48 hours were detected with a 32P-labeled probe and the signals were quantified with a Phosphorimager. Fluorescently labeled 18s rRNA was used for normalization. The signal ratio GFP/rRNA was set to 1 for the non-induced samples. The parental AnTat1.1 13–90 cell line served as a control. (TIF) [file ppat.1006324.s005.tif]

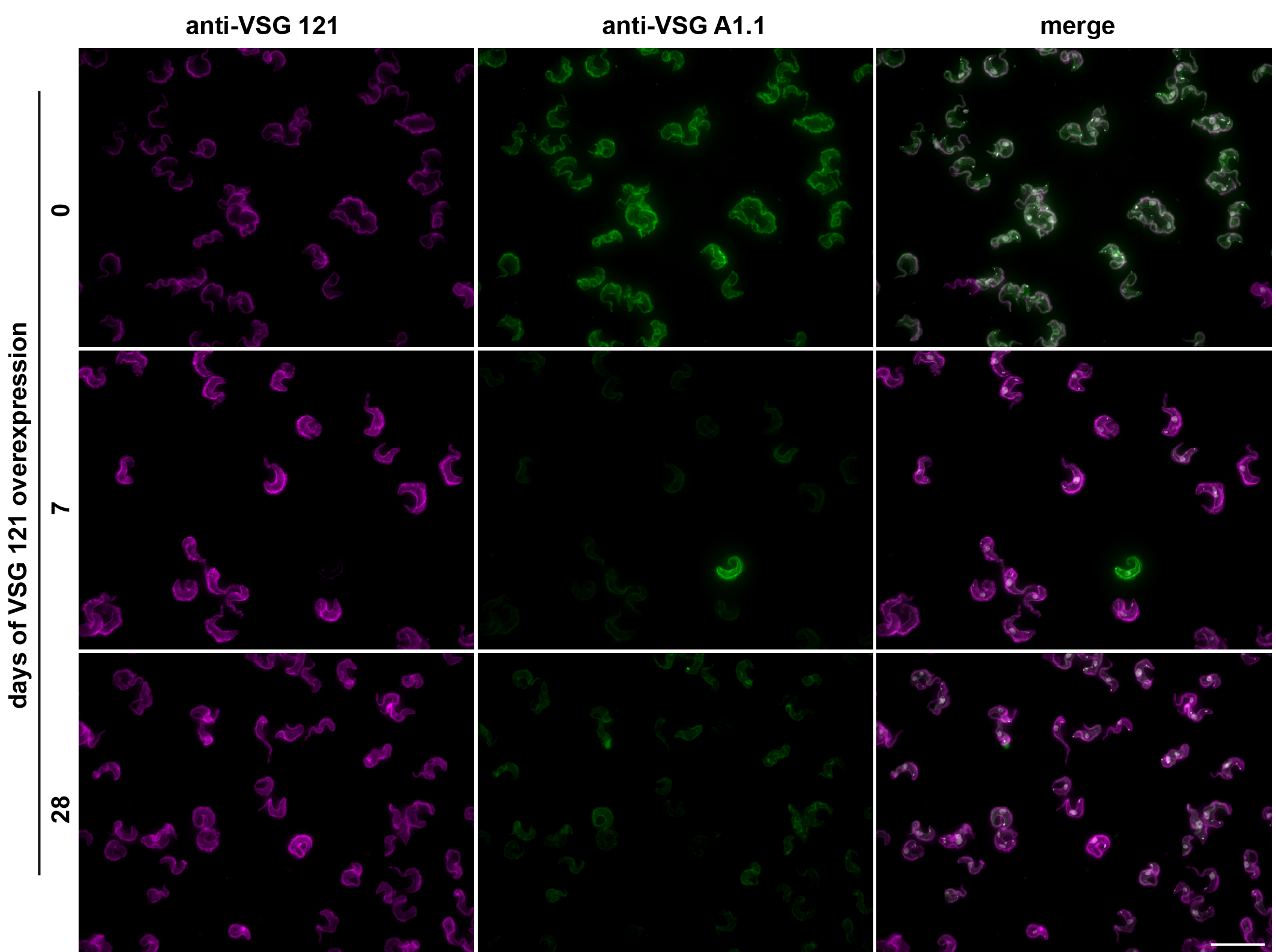

Supplement: S6 Fig — Immunofluorescence analysis of a proliferating clone of the GFP:PAD1UTR reporter cell line using antibodies against the ectopic VSG 121 (magenta, left) and the endogenous VSG A1.1 (green, middle). Non-induced cells (0 days) as well as cells induced for 7 and 28 days were analyzed. The merged antibody signal is shown on the right panel. DNA stained with DAPI (grey) is represented in the merged image only. Scale bar: 20 μm. (TIF) [file ppat.1006324.s006.tif]

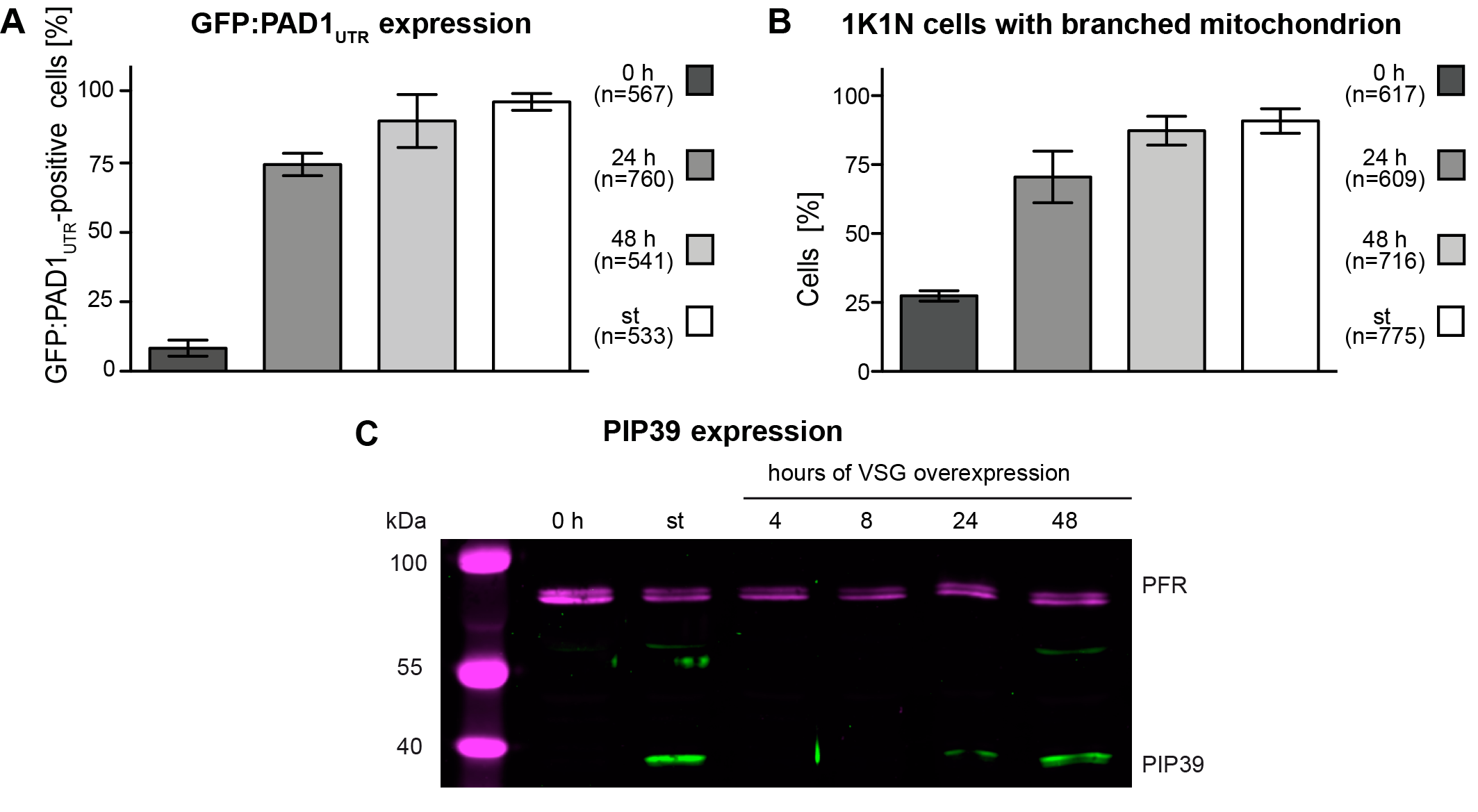

Supplement: S7 Fig — A growth arrested clone of the GFP:PAD1UTR reporter cell line was analyzed. Non-induced slender (0 h) or density-induced stumpy cells (st) of the same clone served as controls. (A) Trypanosomes were microscopically analyzed for the presence of the green fluorescent GFP:PAD1UTR reporter after 24 and 48 hours of ectopic VSG overexpression. Values are given as percentages (± SD) of two experiments (total n > 500). (B) Quantification of 1K1N cells possessing a branched mitochondrion after 24 and 48 hours of ectopic VSG overexpression. The mitochondrion was stained with mitotracker prior to fixation and DAPI staining. Values are given as percentages (± SD) of three experiments (total n > 600). (C) Western blot stained with an antibody against a glycosomal DxDxT class phosphatase (PIP39, green), whose expression increases during density-induced stumpy development (st). PIP39 is upregulated within 48 hours of ectopic VSG overexpression. Detection of paraflagellar rod (PFR) proteins served as a loading control (magenta). (TIF) [file ppat.1006324.s007.tif]

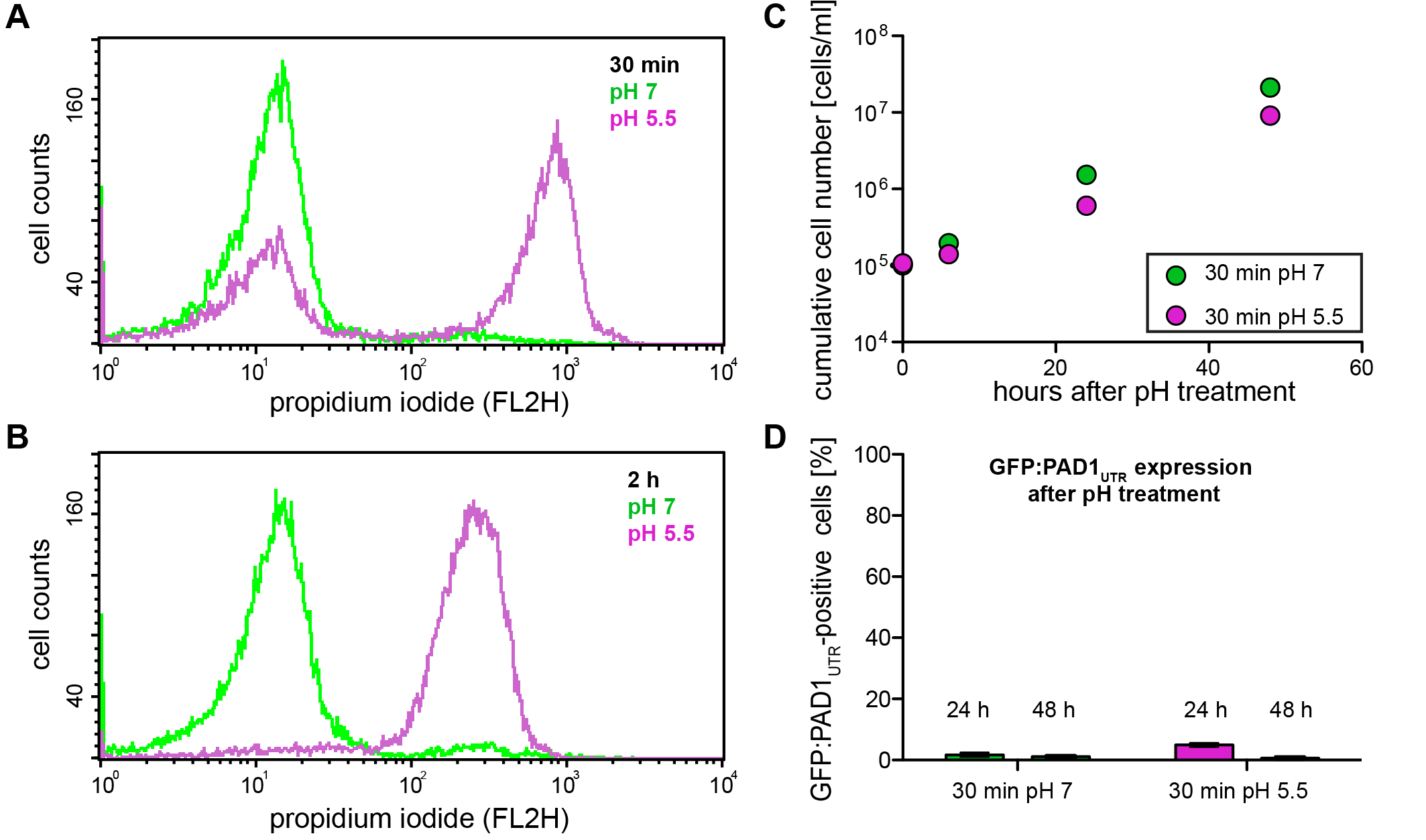

Supplement: S8 Fig — Slender parasites of the GFP:PAD1UTR reporter cell line were incubated in HMI-9 medium at pH 7 or pH 5.5 for (A) 30 minutes or (B) 2 hours. To determine cell viability, the parasites were stained with propidium iodide and analyzed via flow cytometry. Within 30 minutes of incubation at pH 5.5 the majority of the cells had died. After 2 hours no living parasites were detectable. To determine if the cells, which were still viable after 30 minutes of pH-stress, had arrested in the cell cycle and differentiated to the stumpy stage, the culture was washed two-times with TDB and further incubated in HMI-9 at pH 7, supplemented with methylcellulose. (C) Parasite growth was monitored for 48 hours after 30 minutes of treatment at pH 5.5. The mild acid treated cells grew with the same doubling times as the pH 7 control, and hence, had not differentiated. Data are means (± SD) of experiments performed in triplicate. Due to the small standard deviation, the error bars are not visible. (D) The number of GFP:PAD1UTR-positive cells was determined microscopically after 24 and 48 hours of pH treatment for 30 minutes. Values are presented as percentages of cells (± SD) of triplicate experiments (total n > 600 cells). Due to the small standard deviation the error bars are not visible. (TIF) [file ppat.1006324.s008.tif]

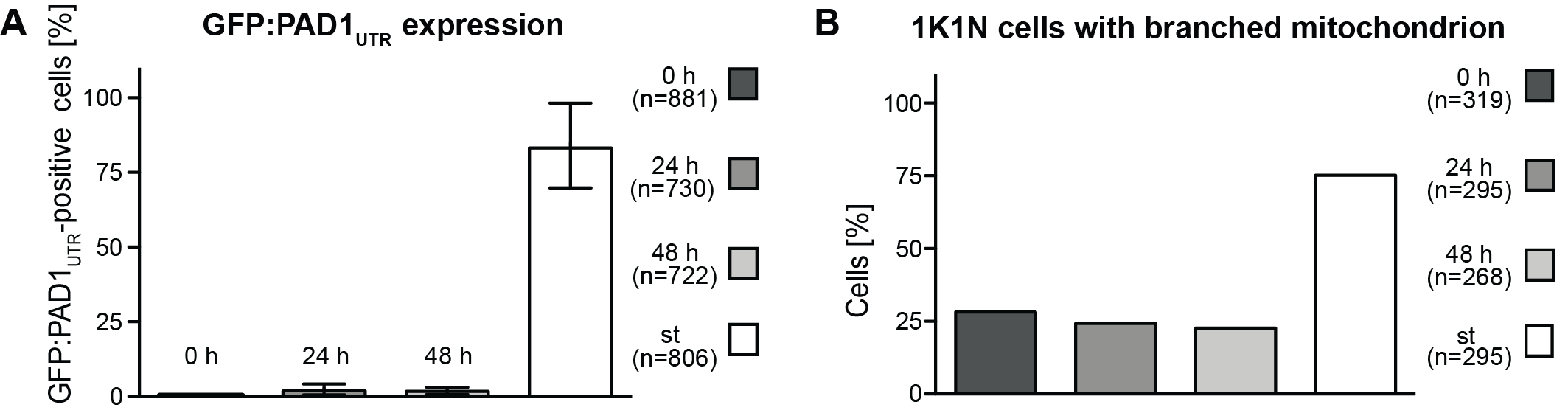

Supplement: S9 Fig — A proliferating clone of the GFP:PAD1UTR reporter cell line was analyzed after 24 and 48 hours of ectopic VSG overexpression. Non-induced slender (0 h) or density-induced stumpy cells (st) of the same clone served as controls. (A) Trypanosomes were microscopically analyzed for the presence of the green fluorescent GFP:PAD1UTR reporter. Values are given as percentages (± SD) of two experiments (total n > 700). (B) Quantification of 1K1N cells possessing a branched mitochondrion. The mitochondrion was stained with mitotracker prior to fixation and DAPI staining. Values are given as percentages (n > 250). (TIF) [file ppat.1006324.s009.tif]

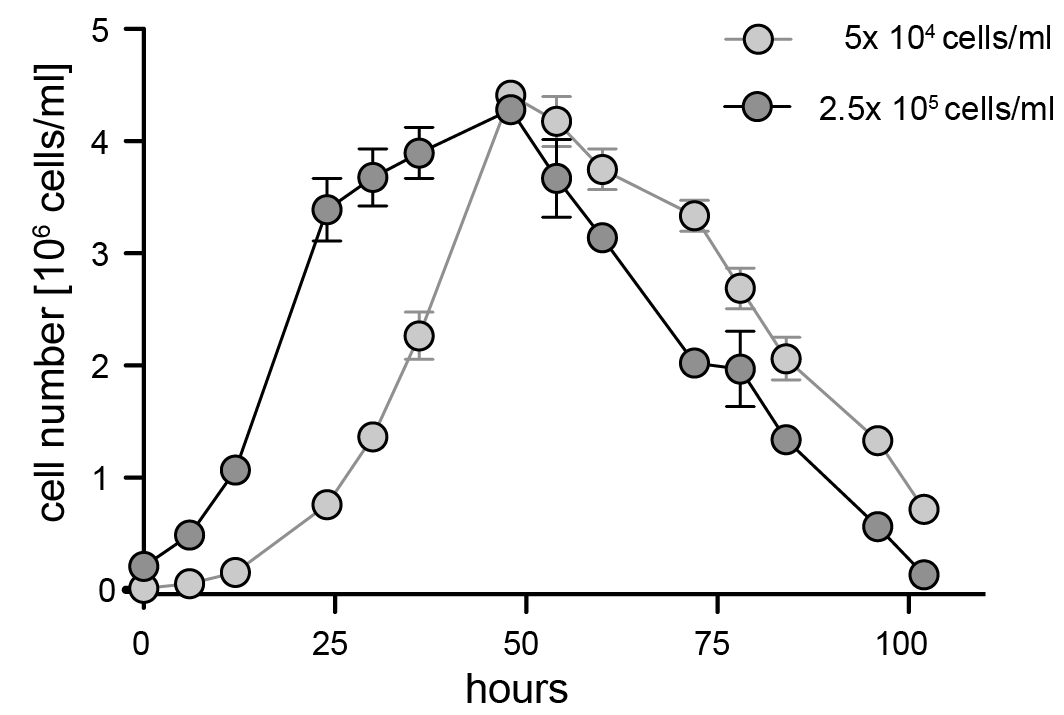

Supplement: S10 Fig — Slender AnTat1.1 parasites were cultivated without dilution. Two different starting cell densities, 5x 10^4^ cells/ml (light grey circles) and 2.5x 10^5^ cells/ml (dark grey circles) were used. Data are means (± SD) of three experiments. (TIF) [file ppat.1006324.s010.tif]

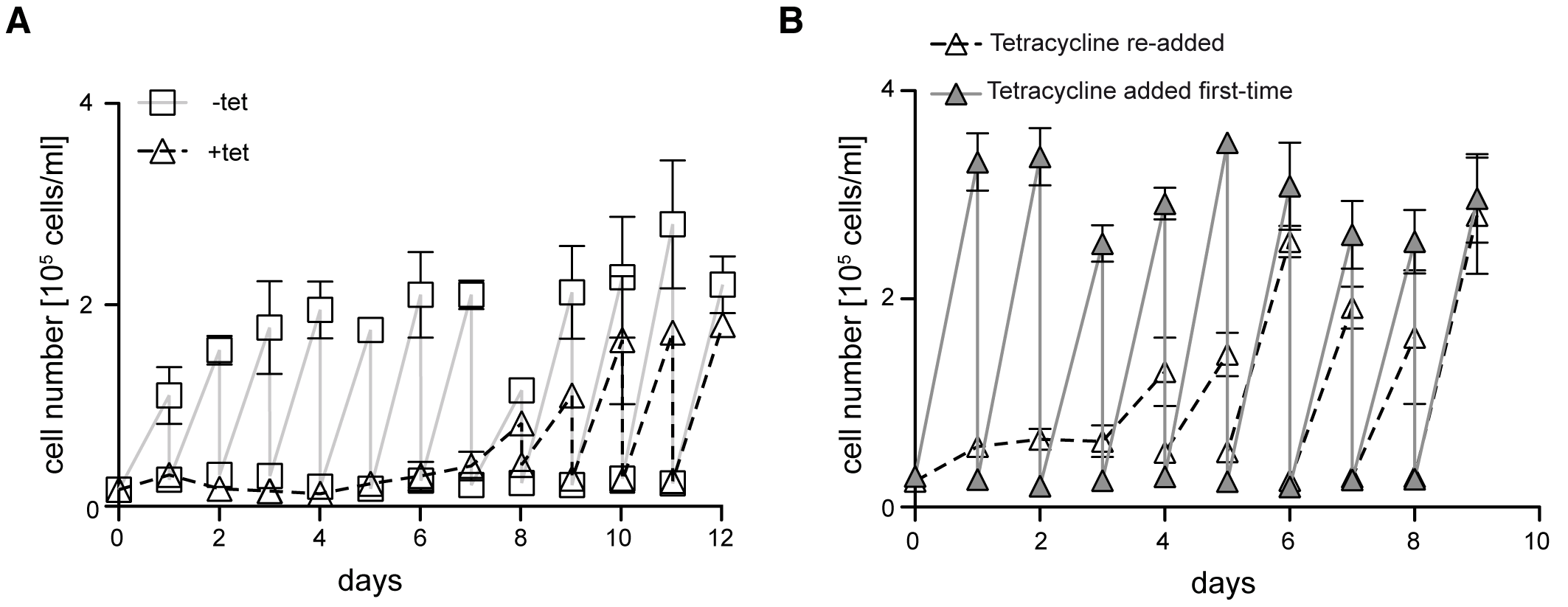

Supplement: S11 Fig — Analyses of a growth arrested clone of the GFP:PAD1UTR reporter cell line that resumed growth. (A) Representative growth curve of parasites induced for the first time (triangles) and non-induced (squares) cells. Data are means (± SD) of two experiments. (B) After 48 hours of ectopic VSG overexpression tetracycline was removed. Then, the parasites were further cultivated without tetracycline for one week. The growth was record once tetracycline was re-added to the culture (grey triangles). Parasites of the same clone, which were cultivated for the same time and had never been induced with tetracycline, were induced for the first time (white triangle) as a control. Data are means (± SD) of three experiments. (TIF) [file ppat.1006324.s011.tif]

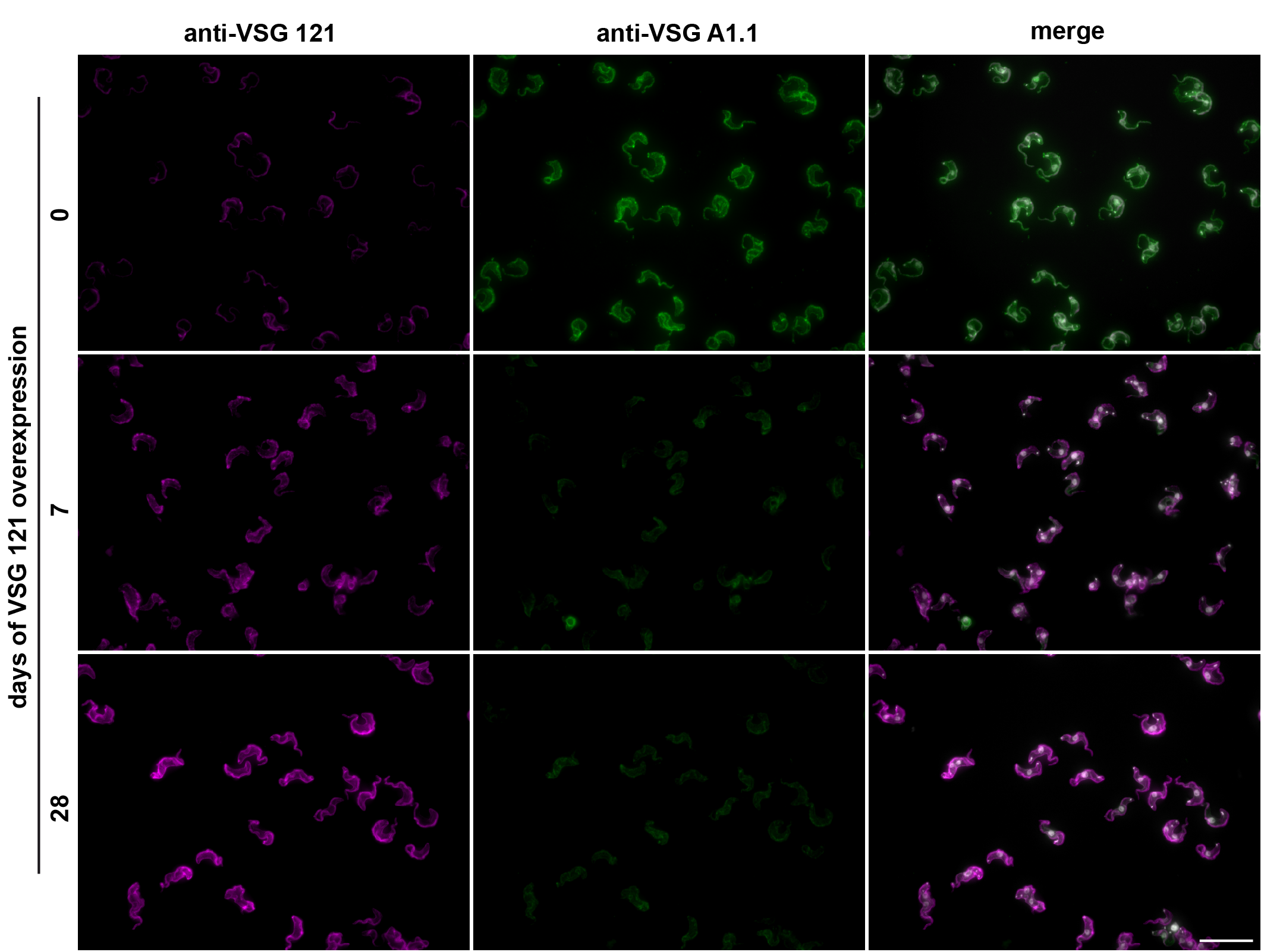

Supplement: S12 Fig — Immunofluorescence analysis of a growth arrested clone of the GFP:PAD1UTR reporter cell line using antibodies against the ectopic VSG 121 (magenta, left) and the endogenous VSG A1.1 (green, middle). Non-induced cells (0 days) as well as cells induced for 7 and 28 days were analyzed. The merged antibody signal is shown on the right panel. DNA stained with DAPI (grey) is displayed in the merged image only. Scale bar: 20 μm. (TIF) [file ppat.1006324.s012.tif]

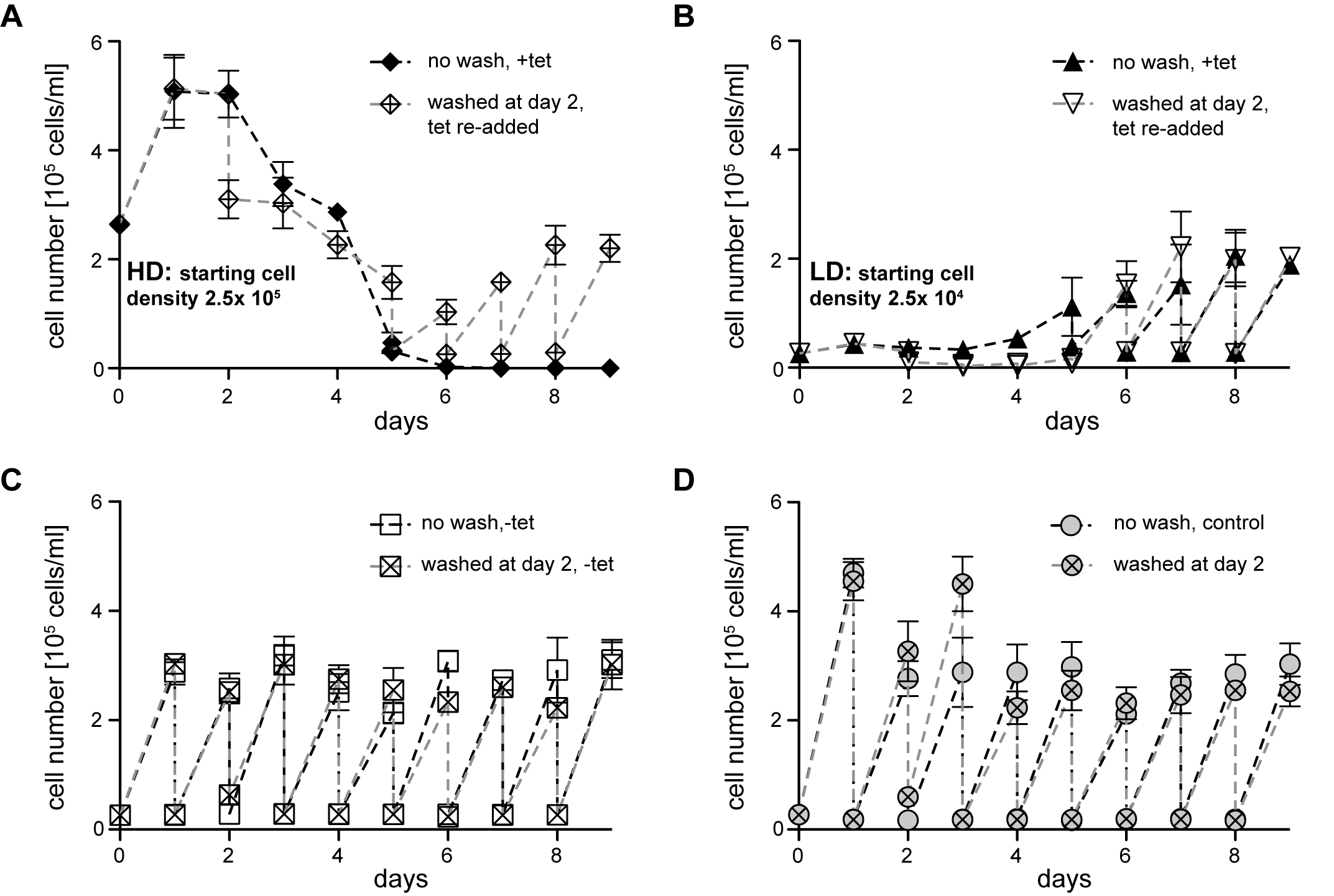

Supplement: S13 Fig — Growth curves were recorded to analyse the impact of SIF on a growth arrested ectopic VSG overexpressor. To determine at which time point the secreted SIF affected the ectopic VSG overexpressors, SIF was removed from the cultures after 2 days of induction by washing (washed at day 2 +tet). Induction was maintained due to the re-addition of tetracycline and cultures were diluted again once they resumed growth. Data are means (± SD) of three experiments. (A) High parasite density (HD, 2.5x 10^5^ cells/ml) allowed the accumulation of SIF during ectopic VSG overexpression. (B) SIF induced stumpy development was prevented at low parasite density (LD, 2.5x 10^4^ cells/ml). (C, D) Non-induced cells (C, -tet) and parasites of the parental AnTat1.1 cell line (D, control) were treated in the same way to verify that washing of the cells did not affect growth. (TIF) [file ppat.1006324.s013.tif]

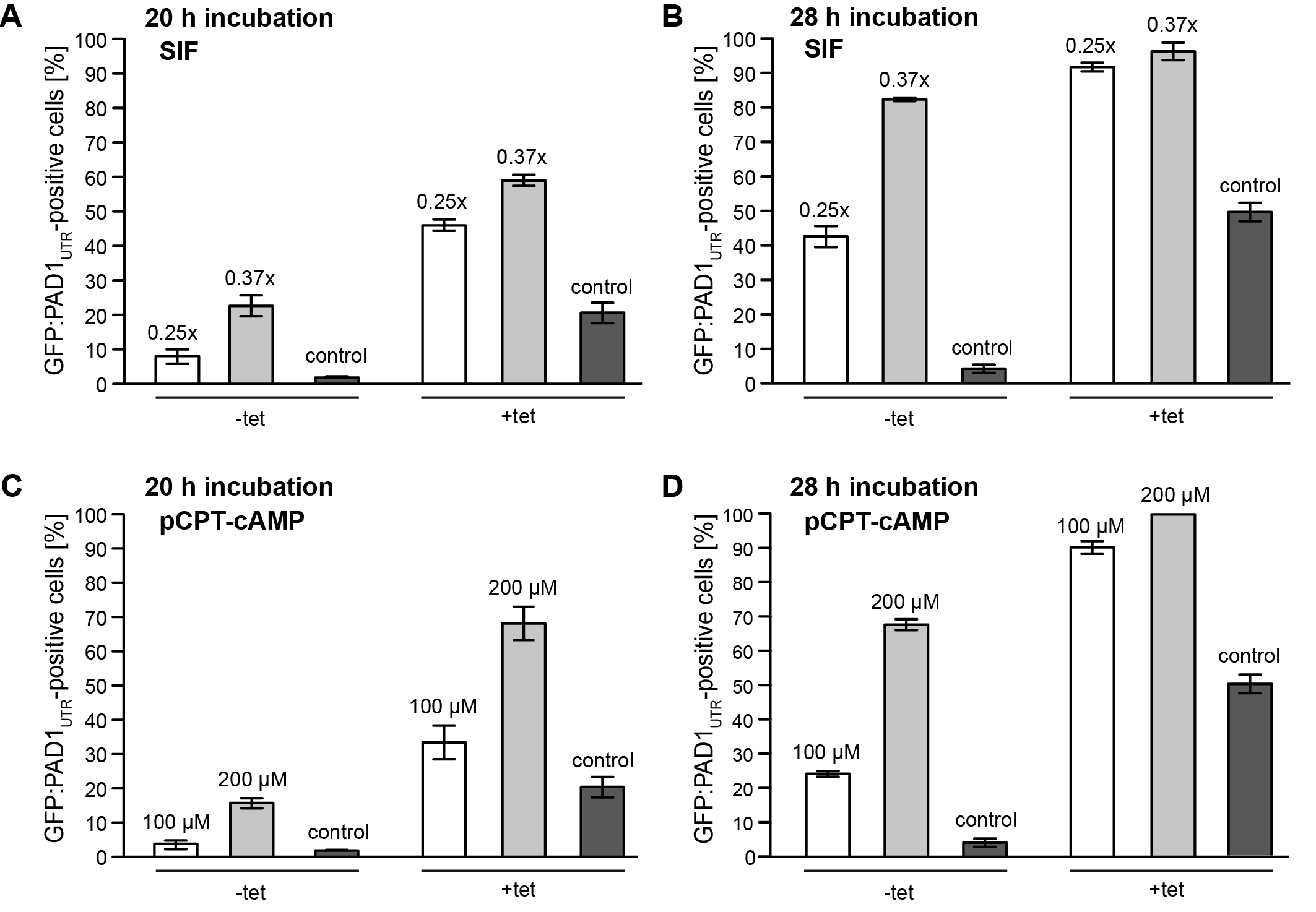

Supplement: S14 Fig — Either non-induced cells (-tet) or cells induced for ES-attenuation (addition of tetracycline, +tet) were challenged at a starting cell density of 1x 10^5^ cells/ml with SIF (A, B) or pCPT-cAMP (C, D). SIF and pCPT-cAMP were both used in two different concentrations that provided a good window for observation. Non-induced and induced cells were analyzed without adding the stumpy differentiation triggers as controls. The amount of GFP:PAD1UTR-positive cells was determined microscopically after 20 and 28 hours of treatment. Values are presented as percentages of cells (± SD) of a triplicate experiment (total n > 600 cells). The data after 20 hours of treatment with 200 μM pCPT-cAMP or 0.25xSIF are merged in Fig 11 with a second experiment. After 20 hours of exposure to the lower SIF concentration almost 6-fold more parasites (0.25x SIF +tet: 46%) expressed the stumpy reporter than in the non-induced cells (0.25x SIF -tet: 8%). With the higher SIF concentration 2-fold more were GFP:PAD1UTR-positive than in the non-induced cells (0.37x SIF +tet: 59%; 0.37x SIF -tet: 22%). In the absence of SIF (control) 2% of the non-induced and 20% of the ectopic VSG overexpressors became stumpy. After 28 hours of incubation with 0.25x SIF, about twice as many cells (91%) expressed the stumpy reporter than in the non-induced parasites (0.25x SIF -tet: 42%). Without additional SIF (control +tet) ES-attenuation alone yielded 50% stumpy cells. Thus, the combination of SIF and ES-attenuation generated a pure stumpy population faster than each trigger alone. This was confirmed with the downstream signal cAMP, with the presence of the additional stumpy-differentiation triggers also causing an increase in the number of stumpy ectopic VSG 121 overexpressors. (TIF) [file ppat.1006324.s014.tif]
